# Suppression of a broad spectrum of liver autoimmune pathologies by single peptide-MHC-based nanomedicines

**DOI:** 10.1038/s41467-019-09893-5

**Published:** 2019-05-14

**Authors:** Channakeshava Sokke Umeshappa, Santiswarup Singha, Jesus Blanco, Kun Shao, Roopa Hebbandi Nanjundappa, Jun Yamanouchi, Albert Parés, Pau Serra, Yang Yang, Pere Santamaria

**Affiliations:** 1 Julia McFarlane Diabetes Research Centre (JMDRC) and Department of Microbiology, Immunology and Infectious Diseases, Snyder Institute for Chronic Diseases and Hotchkiss Brain Institute, Cumming School of Medicine, University of Calgary, Calgary, AB T2N 4N1 Canada; 2 Institut D’Investigacions Biomèdiques August Pi i Sunyer, 08036 Barcelona, Spain; 3 Liver Unit, Hospital Clinic de Barcelona, CIBERehd and University of Barcelona, 08036 Barcelona, Spain; 4 Department of Biochemistry and Molecular Biology, Cumming School of Medicine, University of Calgary, Calgary, AB T2N 4N1 Canada

**Keywords:** Nanoparticles, Peripheral tolerance, Autoimmunity, Immunotherapy, Autoimmune hepatitis

## Abstract

Peptide-major histocompatibility complex class II (pMHCII)-based nanomedicines displaying tissue-specific autoantigenic epitopes can blunt specific autoimmune conditions by re-programming cognate antigen-experienced CD4+ T-cells into disease-suppressing T-regulatory type 1 (TR1) cells. Here, we show that single pMHCII-based nanomedicines displaying epitopes from mitochondrial, endoplasmic reticulum or cytoplasmic antigens associated with primary biliary cholangitis (PBC) or autoimmune hepatitis (AIH) can broadly blunt PBC, AIH and Primary Sclerosing Cholangitis in various murine models in an organ- rather than disease-specific manner, without suppressing general or local immunity against infection or metastatic tumors. Therapeutic activity is associated with cognate TR1 cell formation and expansion, TR1 cell recruitment to the liver and draining lymph nodes, local B-regulatory cell formation and profound suppression of the pro-inflammatory capacity of liver and liver-proximal myeloid dendritic cells and Kupffer cells. Thus, autoreactivity against liver-enriched autoantigens in liver autoimmunity is not disease-specific and can be harnessed to treat various liver autoimmune diseases broadly.

## Abstract (web summary)

Immune response against tissue-specific antigens is a hallmark of autoimmunity. Here the authors show that a single autoantigen-based nanomedicine can ameliorate pathology in a broad range of liver autoimmunity models without impairing host defenses, suggesting organ-wide tolerization.

## Introduction

Nanoparticles (NPs) coated with mono-specific type 1 diabetes (T1D), experimental autoimmune encephalomyelitis (EAE) or collagen arthritis (CIA)-relevant peptide-major histocompatibility complex (MHC) class II (pMHCII) molecules can restore normoglycemia in diabetic animals or motor function in paralyzed mice, and resolve joint swelling and destruction in arthritic mice^1^. pMHCII-NPs directly trigger sustained ligation of cognate antigen receptors on *autoantigen-experienced* FoxP3^–^CD25^–^ T-cells, promoting their differentiation into T-regulatory-type-1 (TR1)-like cell progeny in a phagocyte-independent manner, followed by systemic expansion^1,2^. Consequently, these compounds cannot trigger TR1-like cell formation or expansion in mice that are either disease-free or do not express the cognate autoantigen^1^. These in vivo-expanded TR1-like cells then broadly suppress the polyclonal T-cell responses underlying T1D, EAE, and CIA development in a disease-specific manner, by suppressing local autoantigen presentation and antigen-presenting cell (APC) activation in a cognate antigen-dependent but non-antigen-specific manner (i.e. by recognizing cognate pMHC molecules on costimulation-competent, autoantigen-loaded APCs)^1^.

In autoimmune disorders like T1D, multiple sclerosis (MS) or rheumatoid arthritis (RA), disease results from recruitment of T-lymphocytes and B-lymphocytes recognizing a diverse repertoire of organ-specific autoantigens^3,4^. In other organ-specific autoimmune disorders, such as in liver autoimmune diseases—primary biliary cholangitis (PBC), primary sclerosing cholangitis (PSC) or autoimmune hepatitis (AIH)—the autoimmune response focuses on liver-enriched, non-organ-specific antigens, such as the mitochondrial pyruvate dehydrogenase complex-E2 component (PDC-E2) in PBC; or nuclear, cytoplasmic, or Golgi-enriched proteins, such as F-actin, formimidoyltransferase cyclodeaminase (FTCD), or cytochrome P450 (CYPD2D6) in AIH; or tropomyosin isoform 5 (hTM5) in PSC, among several others^5–7^.

Although AIH, PBC, and PSC are considered as distinct diseases, there is a group of patients presenting with features of both cholestatic liver disease and AIH. Furthermore, PBC is frequently associated with extra-hepatic autoimmune conditions^8^. The existence of these overlap syndromes suggests that activation of T-cells targeting such liver-enriched autoantigens may contribute to various liver autoimmune conditions. In that case, pMHCII-based nanomedicines displaying epitopes from antigens relevant to one disease (e.g. from PDC-E2 in PBC) might be able to trigger the formation and expansion of epitope-specific TR1 cells capable of blunting both the corresponding liver autoimmune disease (e.g. PBC) and other liver autoimmune diseases.

We sought to test this hypothesis by asking if pMHCII-based nanomedicines displaying epitopes from various PBC-relevant or AIH-relevant antigens could blunt liver autoimmunity broadly. We find that pMHCII-based nanomedicines displaying epitopes from various liver-autoimmune disease-relevant antigens can blunt not only the relevant liver autoimmune disease (i.e. PDC-based nanomedicines blunt PBC) but also their irrelevant counterparts (i.e. PSC and AIH in addition to PBC). Remarkably, they do so without impairing the ability of the host to mount antibody responses against exogenous antigens, to clear viral or bacterial infections or to kill metastatic allogeneic tumors. Thus, hepatocyte and cholangiocyte autoimmune insults can readily trigger the stimulation of peripheral T-cells recognizing liver-prevalent self-antigens, and such T-cell responses can be harnessed by pMHCII-based nanomedicines to treat liver autoimmunity broadly.

## Results

### TR1 cell formation and expansion by PBC-relevant pMHCII-NPs

NOD.*c3c4* mice, which carry anti-diabetogenic regions from C57BL/6 chromosomes 3 and 4, spontaneously develop a form of autoimmune biliary disease that resembles human PBC^9^. Like >90% of PBC patients, these mice develop autoreactive T-cell and B-cell responses against the dihydrolipoyl acetyltransferase (E2) and dihydrolipoyl dehydrogenase-binding protein (E3BP) components of the PDC complex^10–12^, leading to biliary epithelial cell destruction, cholestasis, small bile duct proliferation, and liver failure.

We searched for peptides in murine PDC-E2 capable of binding to the NOD/NOD.*c3c4* class II molecule IA^g7^ in silico. IA^g7^-based pMHCs displaying two such epitopes (PDC-E2_166–181_ and PDC-E2_82–96_) or a negative control peptide (the T1D-relevant BDC2.5 mimotope) were purified from culture supernatants of transgenic CHO cells and coated onto functionalized iron-oxide NPs or used to produce pMHC tetramers^1,2^.

**Fig. 1:**
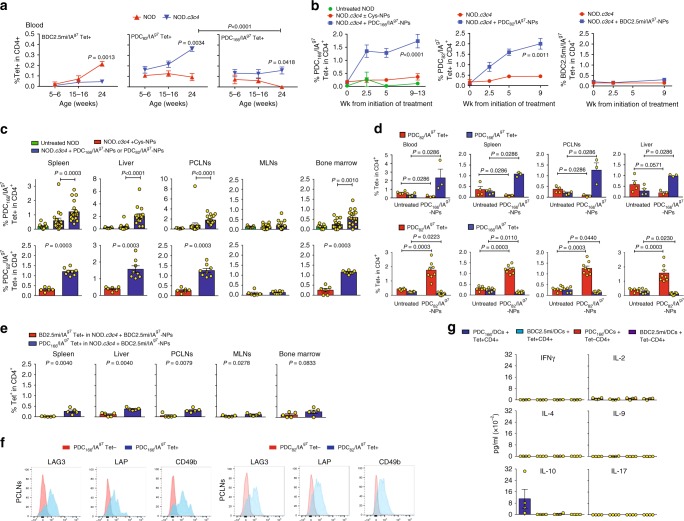
Mitochondrial autoantigen-based pMHCII-NPs expand cognate TR1 cells. **a** Percentages of tetramer+ CD4+ cells in blood of NOD vs. NOD.*c3c4* mice vs. age (4 and 5 mice/strain, respectively). **b** Percentage of tetramer+ CD4+ cells in blood of pMHCII-NP-treated NOD.*c3c4* vs. untreated or control NP-treated NOD or NOD.*c3c4* mice. Data correspond to: 2–5 untreated NOD mice (1 experiment); 2–5 untreated and 15 Cys-NP-treated NOD.*c3c4* mice and 18 PDC_166–181_/IA^g7^-NP-treated NOD.*c3c4* mice (5 experiments); three untreated and five PDC_82–96_/IA^g7^-NP-treated NOD.*c3c4* mice (2 experiments); and 3–5 untreated and 3–5 BDC2.5mi/IA^g7^-NP-treated NOD.*c3c4* mice (2 experiments). **c** Percentages of tetramer+CD4+ cells in various organs from the mice in (**c**) at the end of therapy. **d** Percentages of tetramer+CD4+ cells in lymphoid organs and liver of NOD.*c3c4* mice treated for 9 weeks with PDC_82–96_/IA^g7^-NPs or PDC_166–181_/IA^g7^-NPs. Data correspond to four untreated and three PDC_166–181_/IA^g7^-NP treated, and six untreated and eight PDC_82–96_/IA^g7^-NP-treated NOD.*c3c4* mice. **e** Percentages of tetramer+CD4+ cells in liver and various lymphoid organs in the NOD.*c3c4* mice treated with BDC2.5mi/IA^g7^-NPs from panel **b**. **f** Expression of TR1 markers by PCLN tetramer+CD4+ cells. **g** Cytokine profile of sorted splenic tetramer+CD4+ and tetramer–CD4+ cells upon stimulation with peptide-pulsed DCs. Data correspond to four PDC_166–181_/IA^g7^-NP-treated NOD.*c3c4* mice from three experiments. Data are represented as mean ± SEM. *P* values were calculated via two-way ANOVA (**a** and **b**) or Mann–Whitney *U* (**c**–**e**)

pMHC tetramer staining showed that the peripheral blood of untreated NOD.*c3c4* (but not NOD) mice harbor both PDC-E2_166–181_-reactive and PDC-E2_82–96_-reactive but not BDC2.5mi-reactive CD4+ T-cells, particularly as mice age (Fig. 1a). Treatment of 15-week-old NOD.*c3c4* mice with PDC-E2_166–181_/IA^g7^-NP (twice a week i.v.) triggered the expansion of the PDC-E2_166–181_/IA^g7^ (but not PDC-E2_82–96_/IA^g7^) tetramer+ T-cell pool in peripheral blood (Fig. 1b), spleen, liver, portal/celiac (liver-draining) lymph nodes, and bone marrow, as compared to control NP-treated NOD.*c3c4* littermates (having PBC) or untreated NOD mice (not having PBC) (Fig. 1c, d). In fact, this expansion was associated with significant reductions in the frequencies of endogenous PDC-E2_82–96_/IA^g7^ tetramer+ cells (Fig. 1d). Treatment with T1D-relevant (but PBC-irrelevant) BDC2.5/IA^g7^-NPs did not trigger cognate T-cell expansion (Fig. 1b–e), confirming that pMHC-based nanomedicines exclusively operate on autoantigen-experienced T-cells (BDC2.5-like CD4+ T-cells are not expected to undergo activation by their cognate beta cell autoantigen in the absence of diabetogenic autoimmunity)^1^. As was the case for the TR1-like CD4+ T-cells induced by T1D-relevant pMHC class II-NPs in NOD mice^1^, the PDC-E2_166–181_/IA^g7^ tetramer+ T-cells that expanded in response to PDC-E2_166–181_/IA^g7^-NP treatment were CD25–/FoxP3– and expressed the TR1 markers lymphocyte-activation gene-3 (LAG-3), CD49b (integrin a2 or very-late antigen-2), LAP (transforming growth factor beta latency-associated peptide), program cell death protein-1 (PD1), and inducible T-cell costimulator (ICOS) (Fig. 1f and Supplementary Fig. 1a–c). Although most of the tetramer+ cells expressed LAG-3, expression of CD49b was less penetrant, particularly in the spleen (Supplementary Fig. 1b). This phenotype is similar to that described for the TR1-like CD4+ T-cells arising in NOD mice in response to T1D-relevant pMHC class II-NPs^1^. Increased levels of these markers on tetramer+ CD4+ T-cells isolated from target-organ draining LNs vs. spleen (Supplementary Fig. 1b) is consistent with the positive effects of antigen-induced activation of these cells on TR1 marker expression^1,2^. In addition, the tetramer+ CD4+, but not the tetramer–CD4+ cells from these mice (FACS-sorted) produced IL-10 (but not IFNγ, IL-2, IL-4, IL-9, or IL-17) specifically in response to DCs pulsed with their cognate but not non-cognate peptides ex vivo (PDC-E2_166–181_ vs. BDC2.5mi, respectively; Fig. 1g). Similar results were obtained in mice treated with PDC-E2_82–96_/IA^g7^-NPs, displaying a second PDC-E2-derived IA^g7^-binding peptide (Fig. 1b–d and Supplementary Fig. 1a–c). These TR1-like CD4+ T-cells were not “exhausted” CD4+ T-cells, because unlike CD4+PD1+LAG-3+KLRG1+ (killer-cell lectin-like receptor 1) T-cells isolated from old untreated NOD.*c3c4* mice, PDC-E2_166–181_/IA^g7^ tetramer+CD4+LAG-3+CD49b+ T-cell isolated from PDC-E2_166–181_/IA^g7^-NP-treated animals proliferated in response to stimulation with anti-CD3/anti-CD28 mAb-coated beads ex vivo, without undergoing activation-induced cell death (AICD) (Supplementary Fig. 1d). Thus, as it was the case for T1D, CIA, and EAE-relevant pMHC class II-NPs, PBC-relevant pMHC class II-NPs induce the formation and expansion of TR1-like CD4+ T-cells in vivo.

### TR1 cell-driven reversal of established PBC

**Fig. 2:**
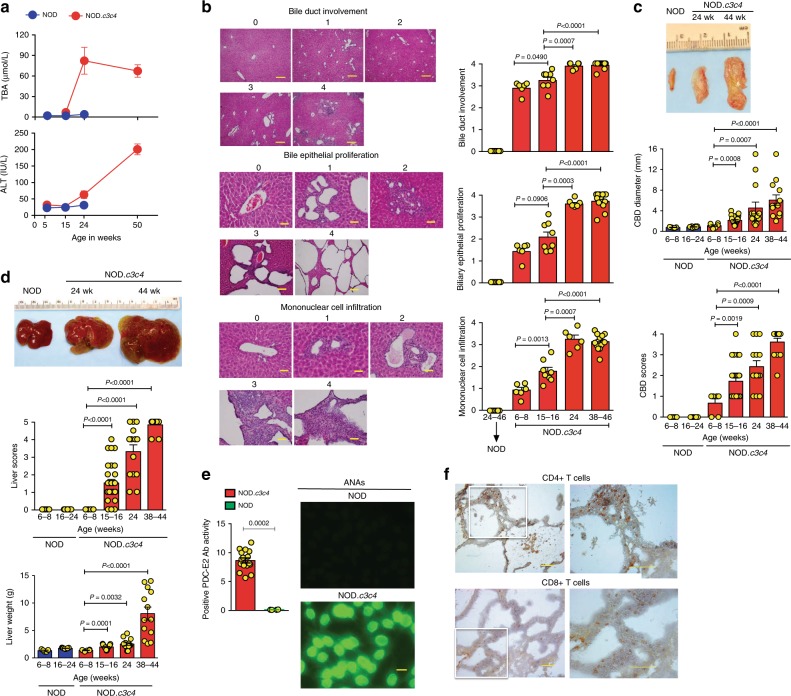
Clinical, phenotypic, immunological, and pathological features of autoimmune cholangitis in NOD.*c3c4* mice. **a** Changes in serum TBA and ALT levels in NOD vs. NOD.*c3c4* mice with age (*n* = 5–6 and 5/time point, respectively). NOD mice could only be evaluated until 24 weeks of age, owing to loss of mice due to diabetes. **b** Microscopic scoring system (left) and progression of microscopic scores of disease with age (right). Data correspond to *n* = 7, 6, 8, 6, and 13 mice from 2 to 3 experiments, from left to right, respectively. Scale bars: top panels: 100 μm; middle and bottom panels: 25 μm**. c** and **d** Representative CBD images and progression of CBD diameter and scores with age (**c**), and representative liver images (**d**, top) and progression of liver scores and weight with age (**d**, bottom). Data in **c** and **d** correspond to *n* = 5, 8, 6, 29, 14, and 13 mice from 2 to 3 experiments, respectively. **e** NOD.*c3c4* but not NOD mice spontaneously develop anti-PDC-E2-specific autoantibodies (left; *n* = 13 and 4 mice/strain type, respectively, from 2 to 3 experiments) and ANAs (right). Photos show representative staining patterns of Hep2 cell nuclei with NOD and NOD.*c3c4* sera at 1:160 dilution. Scale bars: 100 μm. **f** Representative images of liver infiltration by CD4+ and CD8+ T-cells (left: ×20; right: ×40). Scale bars: left photographs: 100 μm; right photographs: 50 μm. Averaged data correspond to the mean  ± SEM. *P* values were compared via Mann–Whitney *U*

NOD.*c3c4* mice display biliary epithelial proliferation, mononuclear cell infiltration of the biliary tree, massive bile duct involvement, and enlargement of the common bile duct (CBD) by 6–8 weeks (Fig. 2b−d). By ~15 weeks, they display increased total bile acid (TBA) levels (Fig. 2a), anti-mitochondrial/PDC-E2-specific autoantibodies (absent in NOD mice; Fig. 2e), and macroscopic signs of liver disease (bile cysts) (Fig. 2d). The severity of these read outs peaks at ~24 weeks (Fig. 2a–d), accompanied by elevated levels of serum alanine aminotransferase (ALT) (Fig. 2a), massive infiltration of the biliary epithelium by both CD4+ and CD8+ T-cells (Fig. 2f), high titers of anti-nuclear autoantibodies (ANAs) (Fig. 2e, right, also absent in NOD mice) and a nearly three-fold increase in liver weight by ~38 weeks of age (Fig. 2d, bottom).

**Fig. 3:**
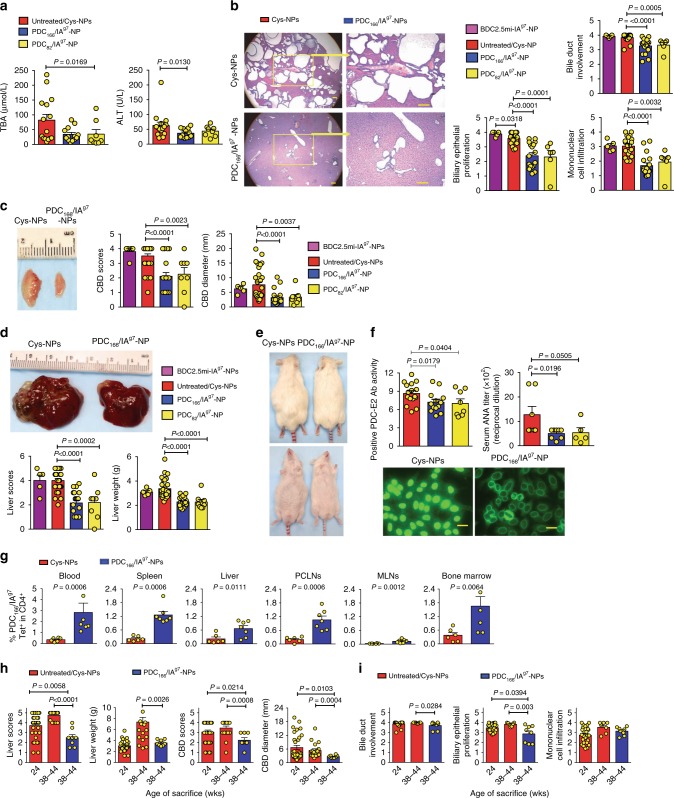
Mitochondrial autoantigen-based pMHCII-NPs blunt PBC in NOD.*c3c4* mice. **a** Changes in serum TBA and ALT levels [*n* = 15, four experiments; *n* = 13, three experiments; and *n* = 8, two experiments (left to right)]. **b** Representative liver micrographs (×4 and ×10) of liver sections from Cys-NP-treated vs. PDC_166–181_/IA^g7^-NP-treated mice (top) and average scores (bottom) [*n* = 5, two experiments; *n* = 20, five experiments; *n* = 17, four experiments; and *n* = 6, two experiments (left to right)]. Scale bars: 100 μm. **c** and **d** Representative common bile duct (CBD) images (**c**, top), average CBD scores/diameters (**c**, bottom), representative livers (**d**, top) and average liver scores/weight (**d**, bottom) [*n* = 5, two experiments; *n* = 30, seven experiments; *n* = 19, four experiments; and *n* = 8, two experiments (left to right)]. **e** Representative images of treated NOD.*c3c4* mice. **f** Anti-PDC-E2 positive antibody activity levels (PAA, see “Methods” for details) and ANA titers (top), and representative images of Hep2 cells stained with diluted (1:160) sera (bottom) (*n* = 16, five experiments; *n* = 13, three experiments; *n* = 8, two experiments, respectively –left–; *n* = 7, 8 and 5, 1–3 experiments, respectively –right–). Scale bars: 100 μm. **g** Percentages of tetramer+ cells in 38–44 week-old mice treated from 24 weeks (*n* = 6 and 7, respectively, two experiments). **h** and **i** Macroscopic (**h**) and microscopic scores (**i**) for the mice in **g** [*n* = 31 (**h**) or 25 (**i**); *n* = 19 (**h**) or 7 (**i**); and *n* = 8 (**h** and **i**) (left to right)]. Data correspond to mean ± SEM. *P* values were calculated via Mann–Whitney *U*

**Fig. 4:**
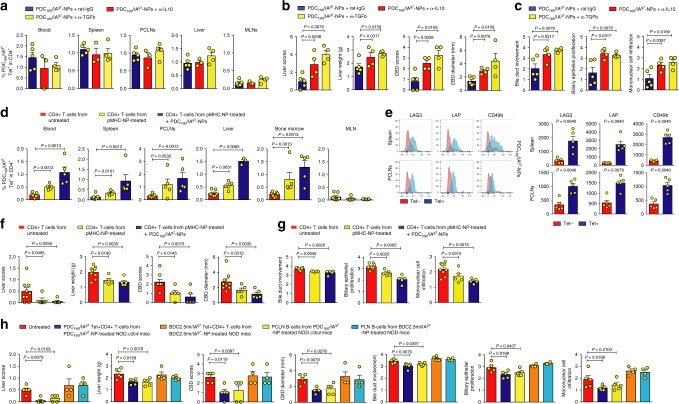
Therapeutic activity of PBC-relevant pMHCII-NPs can be suppressed by IL-10 and TGFβ blockade. **a** Percentages of tetramer+CD4+ T cells in blood and lymphoid organs of NOD.*c3c4.scid* hosts reconstituted with whole splenocytes from untreated NOD.*c3c4* donors a day after transfusion with splenic CD4+ T-cells from untreated or PDC_166–181_/IA^g7^-NP-treated NOD.*c3c4* mice. The hosts were either left untreated or were treated with PDC_166–181_/IA^g7^-NPs. **b** Representative FACS staining histograms (left) and average mean fluorescence intensity values for TR1 markers on tetramer+CD4+ vs. tetramer−CD4+ T-cells (right) of the hosts treated with PDC_166–181_/IA^g7^-NPs. **c** and **d** Macroscopic scores and liver weights (**c**) and microscopic scores (**d**) of the mice studied in **a**. Data in **a**–**d** correspond to means ± SEM, were compared with Mann–Whitney *U*, and correspond to *n* = 9 (blue in **c**) or 7 (blue in **d**), 5 (red in **a**, **c**, and **d**), and 5 mice (green in **a**, **c**, **d**)/treatment group, respectively. **e** Percentages of tetramer+ CD4+ T-cells in mice treated with pMHCII-NPs and rat-IgG (control) or blocking rat mAbs against mouse IL-10 or TGFβ. **f**–**g** Macroscopic (**f**) and microscopic (**g**) scores of the mice studied in **a**. Data in **e**–**g** correspond to *n* = 5, 3–4 and 4 mice/treatment group, respectively. **h** Macroscopic scores, liver weights, and microscopic scores of NOD.*c3c4.scid* hosts reconstituted with whole splenocytes from untreated NOD.*c3c4* donors a day after transfusion of the hosts with: FACS-sorted splenic/PCLN-derived tetramer+CD4+ T-cells from PDC_166–181_/IA^g7^-NP-treated NOD.*c3c4* mice; purified PCLN B-cells from PDC_166–181_/IA^g7^-NP-treated NOD.*c3c4* mice; FACS-sorted splenic/PLN-derived tetramer+CD4+ T-cells from BDC2.5mi/IA^g7^-NP-treated NOD; or purified PLN B-cells from BDC2.5mi/IA^g7^-NP-treated NOD mice. Data in **h** correspond to *n* = 5/group (red, blue and yellow) or *n* = 4/group (orange and cyanine). Data in **a**–**h** correspond to mean ± SEM. *P* values were calculated via Mann–Whitney *U*

Treatment of 15-week-old NOD.*c3c4* mice with PDC-E2_166–181_/IA^g7^-NPs and PDC-E2_82–96_/IA^g7^-NPs, but not BDC2.5/IA^g7^-NPs (displaying a pancreatic beta cell-specific epitope), resulted in significant reductions in all examined biochemical, immunological, macroscopic, and microscopic readouts of PBC (Fig. 3a–f). Similar results were obtained when treatment was initiated at the peak of disease (24 weeks) (Fig. 3g–i and Supplementary Fig. 1e). As was the case for the TR1 cells arising in response to T1D and EAE-specific pMHC class II-NPs ^1^, mAb-based in vivo blockade of IL-10 and TGFβ in pMHC-NP-treated NOD.*c3c4* mice showed that the therapeutic effects of PDC-E2_166–181_/IA^g7^-NPs required these two TR1 cell-derived cytokines (Fig. 4a–c).

To ascertain if the TR1-like CD4+ T-cells arising in these mice in response to pMHC class II-NP treatment were, at least in part, responsible for therapeutic activity, we transferred purified splenic CD4+ cells from PDC-E2_166–181_/IA^g7^-NP-treated or untreated mice into NOD.*scid.c3c4* hosts reconstituted with splenocytes from diseased (untreated) NOD.*c3c4* donors. As shown in Fig. 4d–g, CD4+ cells from PDC-E2_166–181_/IA^g7^-NP-treated mice had significant disease-suppressive activity as compared to CD4+ T-cells from untreated mice, and treatment of the hosts with PDC-E2_166–181_/IA^g7^-NPs enhanced this effect. The specificity of this in vivo disease-suppressive effect was tested by repeating these adoptive transfer experiments but using FACS-sorted PDC-E2_166–181_/IA^g7^ tetramer+ CD4+ T-cells from PDC-E2_166–181_/IA^g7^-NP-treated NOD.*c3c4* donors or control BD2.5mi/IA^g7^ tetramer+ CD4+ T-cells from BDC2.5mi/IA^g7^-NP-treated NOD mice; only the former, but not the latter, were able to suppress disease progression in the hosts as compared to hosts only receiving effector T-cells (Fig. 4h). Thus, these PDC-E2 epitope-based nanomedicines trigger the formation and expansion of cognate TR1-like cells, which then go on to suppress the progression of PBC.

### Suppression of local and proximal APCs

**Fig. 5:**
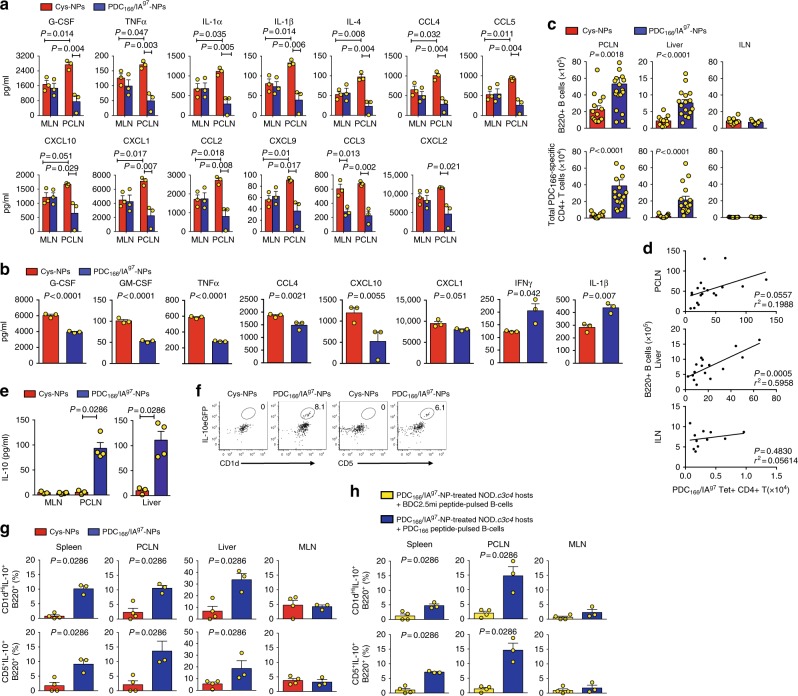
Liver-specific regulatory network formation. **a** Cytokine profile of LPS-challenged CD11b+ cells from PCLNs and MLNs. **b** Cytokine profile of Kupffer cells (only statistically significant analytes shown in **a** and **b**) (*n* = 3/group). **c** Absolute numbers of B-cells (top) and tetramer+ T-cells (bottom) in PCLNs, ILNs, and liver. Data for B-cells correspond to *n* = 15 and 19 (PCLN), *n* = 11 and 11 (ILN), and *n* = 12 and 16, respectively (liver), from 3–5 experiments/organ. Data for tetramer+ cells correspond to *n* = 14 and 19 (PCLN), *n* = 11 and 11 (ILN), and *n* = 15 and 16, respectively (liver), from 3–5 experiments/organ. **d** Correlation between numbers of B-cells and tetramer+ cells in the PCLNs, liver, and ILNs of PDC_166–181_/IA^g7^-NP-treated mice (*n* = 19, 16 and 11; 4, 5 and 3 experiments, respectively). **e** IL-10 secretion by LPS-challenged B-cells from PCLNs, MLNs, and liver (*n* = 4 and 3 mice/treatment type, respectively). **f** Representative FACS plots showing conversion of eGFP– B cells from NOD.*Il10-eGFP* donors into eGFP+/CD5+/CD1d^hi^ Bregs in PDC-E2_166–181_/IA^g7^-NP-treated hosts. **g** Percentages of B cell-to-Breg cell conversion in PDC-E2_166–181_/IA^g7^-NP vs. Cys-NP-treated NOD.*c3c4* hosts, in liver, spleen, PCLNs, and MLNs (*n* = 3 and 4 mice/treatment type, respectively). **h** Percentages of B cell-to-Breg cell conversion in PDC-E2_166–181_/IA^g7^-NP-treated NOD.*c3c4* hosts, in spleen, PCLNs, and MLNs, as a function of the peptide displayed on the B-cell surface (non-cognate: BDC2.5mi, *n* = 4/organ; or cognate: PDC_166–181_, *n* = 3/organ). Data correspond to mean ± SEM. *P* values were calculated via multiple t-test analysis (**a** and **b**), Mann–Whitney *U* (**c**, **e**, **g** and **h**) or Pearson’s correlation test (**d**)

We have previously shown that T1D-relevant antigen-specific TR1-like CD4+ T-cells selectively suppress the proinflammatory and antigen-presenting capacity of pancreatic lymph node-associated APCs by recognizing cognate pMHC class II complexes on autoantigen-loaded APCs draining the pancreas (the source of autoantigenic material)^1,2^. To investigate whether this is also the case for liver autoimmune disease-relevant TR1-like CD4+ T-cells, we compared the cytokine/chemokine profiles of CD11b+ cells purified from the portal/celiac (liver-draining) and mesenteric (non-draining, control) lymph nodes (PCLN and MLN, respectively) of PDC-E2_166–181_/IA^g7^-NP-treated vs. control NP-treated animals (Supplementary Fig. 2a). LPS-challenged CD11b+ cells from the PCLN of control NP-treated animals secreted significantly higher levels of a broad range of pro-inflammatory cytokines and chemokines (*n* = 14/30) than their MLN-associated counterparts, consistent with an increased pro-inflammatory capacity of liver-draining CD11b+ cells in diseased mice (Fig. 5a and Supplementary Fig. 2b). Conversely, the PCLN CD11b+ cells of PDC-E2_166–181_/IA^g7^-NP-treated mice secreted significantly lower levels of these 14 pro-inflammatory mediators than both their mesenteric counterparts and the CD11b+ cells isolated from the PCLNs of control-NP-treated animals. This indicated that, in NOD.*c3c4* mice, treatment with PDC-E2_166–181_/IA^g7^-NPs selectively downregulates the pro-inflammatory capacity of CD11b+ cells draining the liver (Fig. 5a and Supplementary Fig. 2b), presumably because they are loaded with PDC-E2 antigenic material shed from the injured liver and thus display the TR1 cells’ cognate pMHC class II complexes on their surface. pMHC-NP treatment also decreased the secretion of CCL4, IFNγ, and IL-10 by PCLN-associated CD11b+ cells, but this reduction was also seen in MLN-associated CD11b+ cells (Supplementary Fig. 2b). Interestingly, Kupffer cells isolated from PDC-E2_166–181_/IA^g7^-NP-treated mice (Supplementary Fig. 2a) also secreted significantly lower levels of 8 of these 30 mediators (Fig. 5b and Supplementary Fig. 2c). Thus, systemic expansion of PDC-E2-specific TR1 CD4+ cells in NOD.*c3c4* mice is associated with dramatic inhibition of the pro-inflammatory properties of local and proximal PDC-E2 autoantigen-loaded APC types, largely sparing APCs elsewhere.

### Liver B-regulatory (Breg) cell recruitment and formation

The liver and the PCLNs, but not non-liver-draining inguinal LNs (ILNs) of PDC-E2_166–181_/IA^g7^-NP-treated mice harbored significantly higher numbers of PDC-E2_166–181_/IA^g7^-tetramer+ cells and B-cells than those from control-NP-treated animals (Fig. 5c). In addition, the B-cell and tetramer+ T-cell numbers in liver, albeit not PCLNs, were correlated (Fig. 5d). Furthermore, the liver and PCLN, but not the MLN B-cells of PDC-E2_166–181_/IA^g7^-NP-treated mice produced IL-10 in response to LPS, whereas neither the liver nor the PCLN B-cells of control NP-treated animals produced IL-10 (Fig. 5e). Collectively, these observations suggested that, in the liver-draining lymph nodes, PDC-E2_166–181_/IA^g7^-NP-induced TR1-like cells promoted Breg cell formation. These B-cells had disease-specific immunoregulatory activity in vivo, because transfer of purified PCLN B-cells from PDC-E2_166–181_/IA^g7^-NP-treated NOD.*c3c4* mice suppressed the transfer of disease into NOD.*scid.c3c4* hosts reconstituted with splenocytes from diseased NOD.*c3c4* donors. In contrast, the pancreatic lymph node-associated B-cells from BDC2.5mi/IA^g7^-NP-treated NOD mice, which can protect NOD.*scid* mice from diabetes transfer ^1^, had no effect on the ability of splenocytes from sick NOD.*c3c4* mice to transfer PBC into NOD.*scid.c3c4* hosts, suggesting that these B cell-mediated immunoregulatory effects are antigen-specific (Fig. 4h).

To investigate this further, we ascertained the ability of PDC-E2_166–181_/IA^g7^-specific TR1-like cells to promote the differentiation of PDC-E2_166–181_ peptide-pulsed conventional B-cells isolated from NOD.*Il10-eGFP* reporter mice (IL-10/eGFP– B-cells) into CD1d^high^/eGFP+ and CD5+/eGFP+ progeny in vivo (upon adoptive transfer into PDC-E2_166–181_/IA^g7^-NP-treated hosts). As shown in Fig. 5f–g, there was a clear formation of Breg cells in the hosts’ spleen, liver, and PCLNs (containing cognate TR1 cells) but not in the MLNs (lacking cognate TR1 cells). Thus, PDC-E2-specific TR1 cells promote the recruitment and differentiation of conventional B-cells into Breg-like cells. This outcome was driven by cognate pMHC class II interactions between the host’s TR1 cells and the transfused eGFP– B-cells, because it only occurred when the donor B-cells were pulsed with cognate (PDC-E2_166–181_) but not an irrelevant (BDC2.5mi) peptide (Fig. 5h).

### Continued versus intermittent treatment

**Fig. 6:**
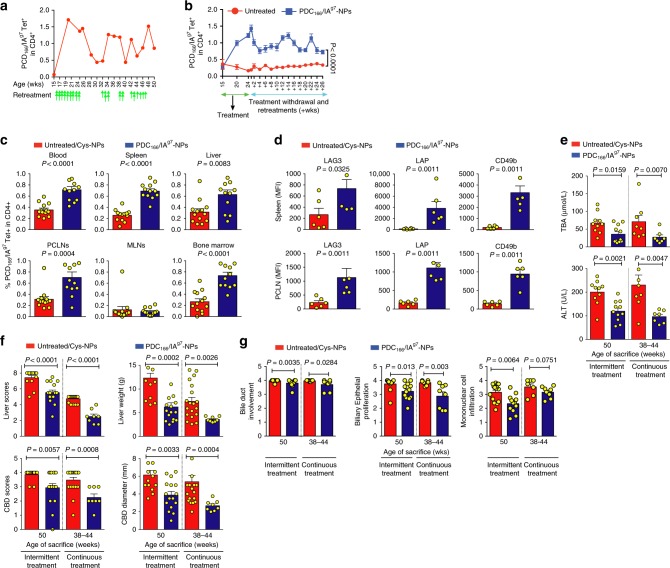
Effects of intermittent vs. continuous therapy. **a** and **b** Changes in the circulating frequency of tetramer+CD4+ T-cells in response to intermittent re-treatment (as a function of circulating tetramer+ T-cell levels). **a** shows profiles of one mouse, where the green arrows indicate the timing of individual doses and double arrowheads indicate that two doses were given in that particular week(s). Treatment was withdrawn at 24 weeks of age. Mice were monitored for persistence of tetramer+ cells in blood once every two weeks. When the percentages of tetramer+ cells fell below 50% of the original values, treatment was re-initiated and when the percentages of tetramer+ cells in blood recovered, treatment was withdrawn again. **b** shows the average values ± SEM corresponding to cohorts of NOD.*c3c4* mice intermittently treated with PDC_166–181_/IA^g7^-NPs (*n* = 19) or left untreated (*n* = 16) after withdrawal of continued therapy (twice a week from 15 to 24 weeks of age). Data are from four experiments. **c** and **d** Percentage of tetramer+CD4+T-cells and mean fluorescence intensity staining for TR1 markers in tetramer+CD4+ T-cells from the mice studied in **a** and **b**. Data in **c** correspond to *n* = 12 pMHCII-NP-treated and 13 untreated mice/organ, respectively, from four experiments. Data in **d** correspond to *n* = 6 mice/group/organ. **e**–**g** Serum TBA and ALT levels (**e**; *n* = 10, 11, 8 and 7, from left to right, respectively), macroscopic (**f;** *n* = 14, 16, 19, and 8, from left to right, respectively) and microscopic (**g;** *n* = 15, 12, 7, and 8 from left to right, respectively) scores from mice treated intermittently (from 15 to 50 weeks of age), or continuously (twice a week from 24 to 38–44 weeks of age). Averaged data correspond the mean ± SEM. *P* values were compared via Mann–Whitney *U* (**c**–**g**) or two-way ANOVA (**b**)

We next examined if the size of the cognate TR1 cell pool arising in blood in response to therapy could be used to gauge the need for re-treatment. As the liver is a large organ, we suspected that the blood-residence time of the PDC-E2-specific TR1 cells in diseased NOD.*c3c4* mice would be significantly shorter than in diabetic NOD mice, where cells can persist in the circulation for months after treatment withdrawal ^1^. The blood tetramer+ T-cell content from most mice declined to ~50% of the original values within 4–6 weeks after treatment withdrawal. Re-treatment rapidly restored these values (Fig. 6a, b). Intermittent therapy given up to 50 weeks of age did not compromise the pharmacodynamic or therapeutic effects of pMHCII-NPs (Fig. 6c–g), as compared to mice treated continuously, supporting the safety of these compounds, even when administered for prolonged periods of time.

### pMHCII-NPs versus the standard of care

Ursodeoxycholic acid (UDCA, a hydrophilic bile acid) is the standard of care for PBC. Although effective in ~50% of patients when given early, it is ineffective at advanced stages of PBC^13^.

**Fig. 7:**
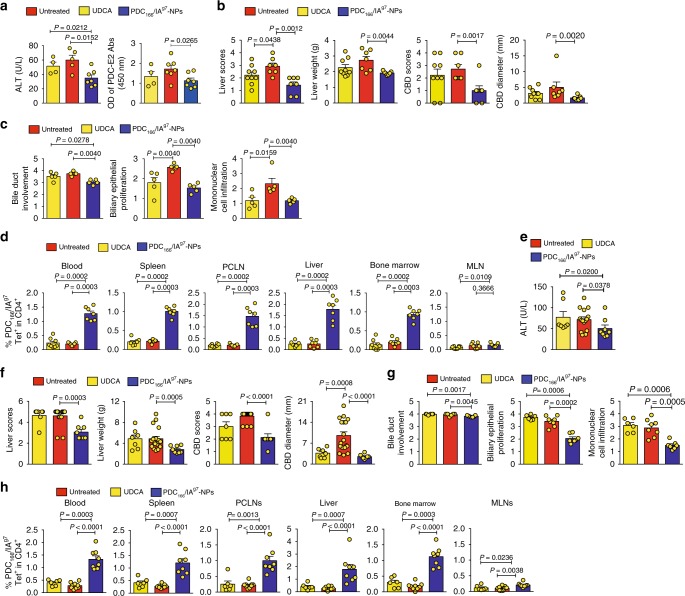
pMHCII-NPs versus the standard of care in PBC. **a**–**c** Effects of treatment with PDC_166–181_/IA^g7^-NPs or the standard of care in PBC (UDCA) on serum ALT (**a;** *n* = 4, 5, and 7, from left to right, respectively), macroscopic (**b;** *n* = 9, 7 and 7, from left to right, respectively) and microscopic (**c;** *n* = 5/group) disease scores, when treatment is initiated early on in the disease process (from 6 to 20 weeks of age; twice a week for PDC_166–181_/IA^g7^-NPs or continuously for UDCA). **d** Percentages of tetramer+CD4+ T-cells in the mice studied in **a**–**c**. Data correspond to *n* = 8, 7 and 7 mice from left to right, respectively. Data in **a**–**d** are from 2 to 3 experiments/group. **e**–**g** Effects of treatment with PDC_166–181_/IA^g7^-NPs or UDCA on serum ALT levels (**e**; *n* = 7, 15, and 8, from left to right, respectively), macroscopic (**f**; *n* = 7, 8 and 18, from left to right, respectively) and microscopic (**g**; *n* = 6, 8 and 7 from left to right, respectively) disease scores, when treatment is initiated at advanced stages of the disease process (from 24 to 33 weeks of age; twice a week for PDC_166–181_/IA^g7^-NPs or continuously for UDCA). **h** Percentages of tetramer+CD4+ T-cells in the mice studied in **e**–**g**. Data correspond to *n* = 6, 9 and 8 mice from left to right, respectively. Data in **e**–**h** are from 2 to 3 experiments/group. Averaged data correspond the mean ± SEM. *P* values were compared via Mann–Whitney *U*

Intake of UDCA-supplemented chow by 6-week-old NOD.*c3c4* mice for 14 weeks had a small therapeutic effect on the progression of PBC, as manifested by reductions in liver scores and liver weight, bile duct proliferation and mononuclear cell infiltration, albeit not serum ALT or TBA levels, CBD scores, CBD diameter, or bile duct involvement (Fig. 7a–d). However, when UDCA was given at 24 weeks, it had none of these effects, except for a very significant reduction in CBD diameter, possibly because of its anti-cholestatic effects (Fig. 7e–g). In contrast, PDC-E2_166–181_/IA^g7^-NPs had substantial therapeutic effects in both 6-week-old and 24-week-old animals (Fig. 7a–h).

### Therapeutic effects in another PBC model

The NOD.*c3c4* model does not fully recapitulate the immunopathology of human PBC, characterized by female prevalence, progression to liver fibrosis, and absence of liver cyst formation. B6 mice carrying a deletion of the IFNγ 3′-untranslated region adenylate uridylate-rich element (ARE) (ARE-Del^+/–^) have a dysregulated *Ifng* locus, and develop a form of PBC that, like the human disease, primarily affects females and is associated with up-regulation of TBA, production of anti-PDC-E2 autoantibodies, portal duct and lobular liver inflammation, bile duct damage and fibrosis^14^. Treatment of female (NODxB6.IFNγ ARE-Del^–/–^) F1 mice with PDC-E2_166–181_/IA^g7^-NPs triggered TR1 cell formation/expansion and suppressed the upregulation of TBA and ALT levels, liver inflammation and fibrosis, as compared to mice treated with control NPs (Supplementary Fig. 3a–d). Similar results were obtained in B6.IFNγ ARE-Del^–/–^ mice treated with NPs displaying an IA^b^-binding PDC-E2-derived epitope (PDC-E2_94–108_/IA^b^-NP) (Supplementary Fig. 3e, f), indicating that the therapeutic activity of these compounds is not a peculiarity of the NOD genetic background or its unique MHC class II molecule.

### Humanized mice with PBC

DRB4*0101 and DRB1*0801 have been associated with PBC in some studies^15^. HLA-DRB-typing of 154 PBC patients from our cohort indicated that 61.7% carried DRB4*01 and 14% DRB1*0801.

Several T-cell epitopes from PDC-E2 binding to two of these HLA-DRB types have been described^12,16^. We compared the ability of PDC-E2_122–135_/DRB4*0101-NPs, PDC-E2_249–262_/DRB4*0101-NPs, and PDC-E2_629–643_/DRB1*0801-NPs to expand cognate TR1-like CD4+ T-cells in NOD.*scid/Il2rg*^–/–^ (NSG) hosts reconstituted with PBMCs from 11 DRB4*0101+ and 5 DRB1*0801+ PBC patients (PBL-NSG mice, Supplementary Fig. 4 and Supplementary Table 1). Supplementary Fig. 4a–c show that the hosts were engrafted with hCD45+ cells containing hCD4+ cells and hCD19+ cells, but no mCD4– cells, and that their mCD45+ cells lacked mCD4+ or hCD4+ cells, as expected. We saw expansion of tetramer+CD49b+LAG-3+CD4+ T-cells in the spleen and/or liver and LNs from 5/6 PBL-NSG mice treated with PDC-E2_122–135_/DRB4*0101-NPs, 4/6 PBL-NSG mice treated with PDC-E2_249–262_/DRB4*0101-NPs and 2/5 PBL-NSG mice treated with PDC-E2_629–643_/DRB1*0801-NPs (Supplementary Table 1). Treated responsive mice had significantly higher percentages and absolute numbers of tetramer+ cells in spleen, liver, lymph nodes, and/or bone marrow (Supplementary Fig. 4d, e) than untreated or unresponsive mice, and these cells expressed the TR1 markers CD49b and LAG-3 (Supplementary Fig. 4f).

### Disease versus organ specificity

Given that PDC-E2 is an autoantigen expressed in virtually all cell types, our results begged the question of whether PBC-relevant nanomedicines (i.e. PDC-E2_166–181_/IA^g7^-NP) are disease-specific or not.

PSC is a chronic cholestatic disease characterized by inflammation of intra-hepatic and extra-hepatic bile ducts leading to a fibro-obliterative cholangitis with periductal fibrosis around medium and large bile ducts and degenerative changes of the biliary epithelium, in the absence of anti-mitochondrial autoantibodies^17^. *Abcb4* knockout mice (lacking the multidrug resistance protein 3) develop a form of cholangitis similar to human PSC that is caused by impaired biliary phospholipid secretion^17^.

AIH is characterized by a portal mononuclear cell infiltration of the liver parenchyma that is associated with presence of ANAs and/or smooth muscle (AIH type 1) or anti-liver kidney microsomal or anti-liver cytosol type 1 autoantibodies, which target the microsomal cytochrome CYP2D6 or FTCD, respectively (AIH Type 2)^18^. Recently, it has been shown that infection of NOD mice with a replication-defective adenovirus encoding human FTCD (Ad-hFTCD) triggers a form of chronic AIH that resembles AIH type 2^19^.

**Fig. 8:**
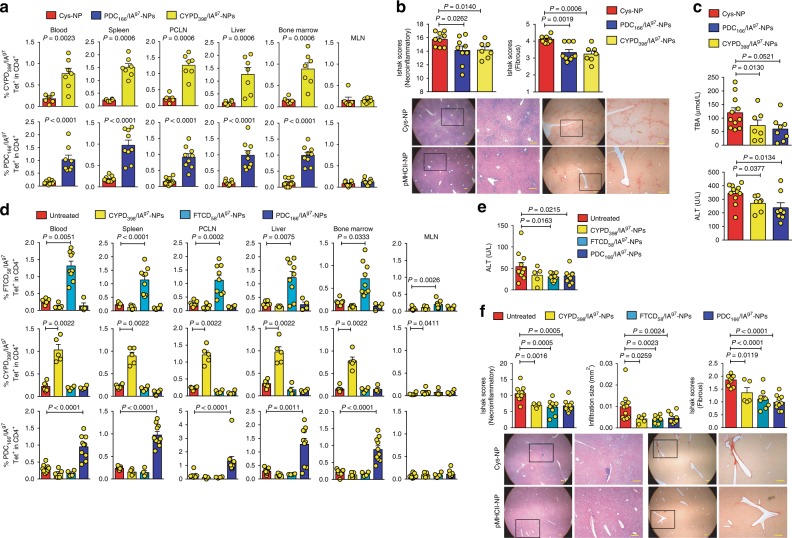
PBC-relevant and AIH-relevant pMHCII-NPs blunt PBC, PSC, and AIH in a disease non-specific manner. **a** Percentage of tetramer+CD4+ T-cells in NOD.*Abcb4*^–/–^ mice in response to Cys-NP, PDC_166–181_/IA^g7^-NP, or CYPD_398–412_/IA^g7^-NP therapy [2 doses/week for 5 weeks starting at 6 weeks of age; *n* = 9–11 (bottom), or 6–7 (top), respectively, from 2 to 3 experiments]. **b** Average PSC scores (top) and representative H&E-stained or Picrosirius-Red-stained liver sections (bottom) from the mice in **a** (*n* = 9, 9 and 7 mice/NP type, respectively; 2–3 experiments). Scale bars: 100 μm. **c** Serum TBA and ALT levels in the mice in **a** (*n* = 11, 8 and 7 mice/NP type, respectively; 2–3 experiments). **d** Percentage of tetramer+ cells in NOD mice infected with Ad-hFTCD and treated with CYPD_398–412_/IA^g7^-NPs (*n* from top, middle, and bottom panels = 5, 5, and 5, respectively), FTCD_58–72_/IA^g7^-NPs (*n* = 9, 4, 4, respectively) or PDC_166–181_/IA^g7^-NPs (*n* = 4, 4, 10, respectively) or left untreated (*n* = 7, 6, 9, respectively). **e** Serum ALT levels in the mice in **d** [*n* = 11, 5, 9, and 10 mice (left to right); 2–3 experiments]. **f** AIH histopathological scores (top) and representative H&E-stained and Picrosirius-Red-stained liver sections (bottom) for the mice in **e**. Scale bars: 100 μm. Data correspond to the mean ± SEM. *P* values were calculated via Mann–Whitney *U*

We reasoned that the large bile duct and parenchymal liver damage that underlie PSC and AIH, respectively, might trigger the release of PDC-E2, CYP2D22 (the mouse CYP2D6 ortholog, herein referred to CYPD) and FTCD, and the priming of autoreactive CD4+ T-cells capable of responding to the corresponding pMHC-NPs. To investigate this, we tested the ability of PDC-E2_166–181_/IA^g7^-NPs (PBC-relevant) and CYPD_398–412_/IA^g7^-NPs (AIH-relevant) to expand cognate TR1-like CD4+ T-cells and ameliorate PSC in NOD.*Abcb4*^–/–^ mice. Remarkably, both PDC-E2_166–181_/IA^g7^ and CYPD_398–412_/IA^g7^-NPs expanded cognate TR1 CD4+ T-cells (Fig. 8a and Supplementary Fig. 5) and reduced liver necroinflammation and fibrosis (Fig. 8b), as well as serum ALT and TBA levels (Fig. 8c), as compared to controls. Likewise, PDC-E2_166–181_/IA^g7^-NPs (PBC-relevant) and both mFTCD_58–72_/IA^g7^-NPs and CYPD_398–412_/IA^g7^-NPs (AIH-relevant) triggered cognate TR1 cell expansion (Fig. 8d and Supplementary Fig. 6a, b) and significant reductions in ALT levels, hepatocyte necrosis, liver inflammation, and liver fibrosis in Ad-hFTCD-infected NOD mice (Fig. 8e, f).

**Fig. 9:**
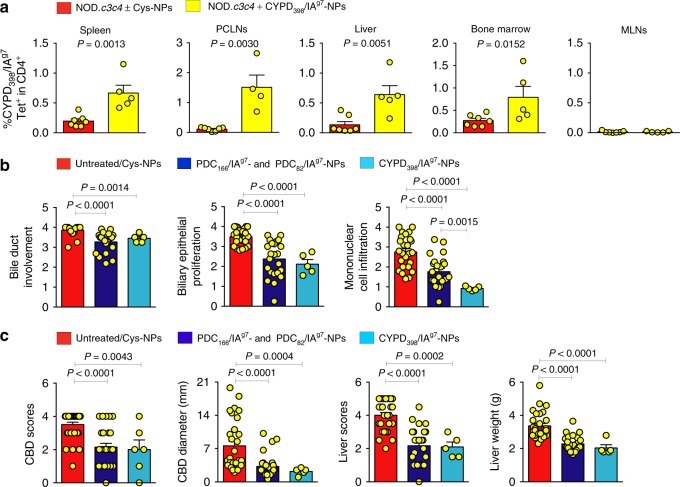
AIH-relevant pMHCII-NPs are as efficient as PBC-relevant pMHCII-NPs to blunt PBC in NOD.*c3c4* mice. **a** Percentage of tetramer+ cells in untreated/Cys-NP-treated (*n* = 7), or CYPD_398–412_/IA^g7^-NP-treated mice (*n* = 5). **b** and **c** Average microscopic (**b**) or macroscopic (**c**) liver scores for the mice treated with PDC_166–181_/IA^g7^-NPs or PDC_82–96_/IA^g7^-NPs (*n* = 23, five experiments for **b** and *n* = 27, six experiments for **c**) vs. CYPD_398–412_/IA^g7^-NPs (*n* = 5, one experiment), as compared to untreated/Cys-NP-treated mice (*n* = 20, six experiments for **b** and *n* = 30, seven experiments for **c**). Data correspond to the mean ± SEM. *P* values were calculated via Mann–Whitney *U*

This ability of ubiquitous autoantigen-based pMHC-nanomedicines to blunt liver autoimmunity in an organ-specific rather than disease-specific manner also occurred in NOD.*c3c4* mice treated with CYPD_398–412_/IA^g7^-NPs (Fig. 9a–c). In fact, the latter were as efficient as PDC-E2_166–181_/IA^g7^-NPs at expanding cognate TR1 cells (Fig. 9a) and blunting PBC in 15-week-old mice (Fig. 9b, c).

Collectively, these observations suggest that hepatocyte (AIH) and bile duct epithelial (PBC and PSC) damage in liver autoimmunity results in the delivery of significant amounts of liver-prevalent autoantigens, including PDC-E2, CYPD2D6, and FTCD to local and proximal APCs. In turn, this enables autoreactive CD4+ T-cell priming (a sine qua non requirement for pMHC-NP-induced TR1 cell formation^1^), cognate TR1 cell generation by pMHC-NPs, Breg cell formation, and suppression of the pro-inflammatory capacity of local and proximal autoantigen-loaded APCs.

### Normal immunity is spared

**Fig. 10:**
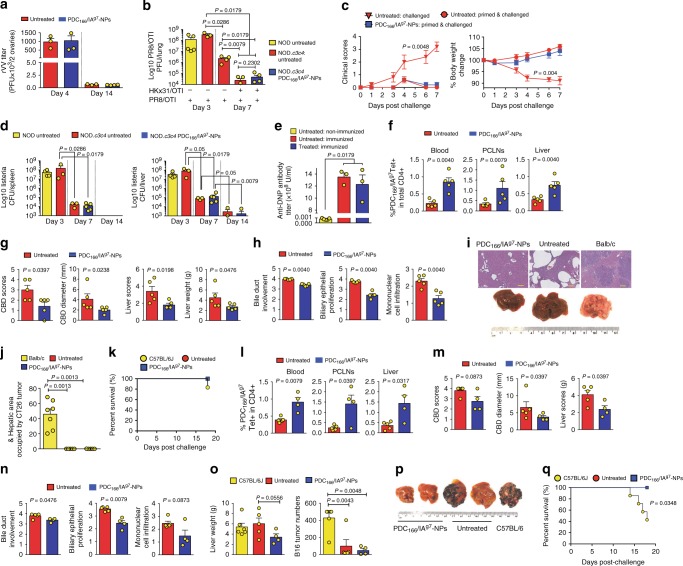
Liver disease-relevant pMHCII-NPs spare general immunity. **a** rVV titers in ovaries 4 and 14 days after rVV infection (*n* = 3–4/group). **b** Influenza viral titers in lungs 3 and 7 days after infection with PR8 with or without HKx31 priming (*n* = 5, 3, 4, 5 and 5; left to right). **c** Clinical scores and body weight (*n* = 5 mice/group). **d** Colony-forming units of *L. monocytogenes* in the spleen and livers 3, 7, and 14 days after infection (*n* = 4, 3, 3, 5, 3 and 4; left to right). **e** Serum anti-DNP antibody titers upon KLH–DNP immunization (*n* = 3/group). **f**–**k** Percentages of tetramer+ cells (**f**), macroscopic PBC scores and liver weight (**g**), microscopic PBC scores (**h**), liver images (**i**), microscopic tumor scores (**j**), and survival rates (**k**) of untreated vs. PDC_166–181_/IA^g7^-NP-treated NOD.*c3c4* (*n* = 5/group) or untreated Balb/c mice (*n* = 7) challenged with CT26 cells (overlapping 100% survival in untreated vs. PDC_166–181_/IA^g7^-NP-treated mice). Scale bar in (**i**): 100 μm. **l**–**p** Percentages of tetramer+ cells (**l**), macroscopic (**m**), and microscopic PBC scores (**n**), and liver weight and metastasis number (**o**), and liver images (**p**) of untreated vs. PDC_166–181_/IA^g7^-NP-treated NOD.*c3c4* (*n* = 5 and 4) or untreated B6 mice (*n* = 6) challenged with B16/F10 cells. **q** Survival rates of the mice in **l**–**p** (overlapping 100% survival in untreated vs. PDC_166–181_/IA^g7^-NP-treated mice). Data correspond to mean ± SEM. *P* values were compared via Mann–Whitney *U* except for **k**, **q** (log-rank), or **c** (two-way ANOVA)

We next investigated if persistent expansion of PDC-E2_166–181_/IA^g7^-specific TR1 cells results in suppression of normal immunity against infection and cancer. Cohorts of NOD.*c3c4* mice received doses of PDC-E2_166–181_/IA^g7^-NPs or control NPs twice a week for 9 weeks. At the end of therapy, the mice were given an i.v. injection of recombinant vaccinia virus. The viral titers in the ovaries of females 14 days after infection were similar in both cohorts of mice and substantially lower than those found at the peak of infection, indicating that pMHCII-NP therapy did not impair cellular immunity against the virus-infected cells (Fig. 10a).

To probe this further, we infected PDC-E2_166–181_/IA^g7^-NP-treated or untreated NOD.*c3c4* mice with a laboratory strain of influenza (HKx31 –H3N2–) i.p. to induce heterologous (shared) immunity against a subsequent lethal infection with an H1N1 strain of Influenza (PR8) given via the intranasal route. As shown in Fig. 10b, c, pMHCII-NP treated NOD.*c3c4* mice mounted protective immunity against the PR8 infection, as documented by decreased viral load in lung tissue and clinical signs of active infection, despite systemic presence of cognate TR1-like CD4+ T-cells (Supplementary Fig. 7a).

Similar results were obtained in mice infected with the intracellular pathogen *Listeria Monocytogenes* (LM). LM-infected PDC-E2_166–181_/IA^g7^-NP-treated and untreated NOD.*c3c4* mice were equally efficient at clearing the bacteria from both the spleen and liver, consistent with unimpaired immunity against this intracellular pathogen (Fig. 10d and Supplementary Fig. 7b). This outcome was also true when the mice were infected with LM shortly before initiation of pMHC-NP treatment. As shown in Supplementary Fig. 7c–f, diseased NOD.*c3c4* mice infected with LM immediately before initiation of treatment and treated for 5 consecutive weeks had, at the end of follow up, significantly reduced disease scores, as well as numbers of LM colony forming units (cfu) in both the liver and spleen. Importantly, this reduction in splenic and liver LM cfu was similar in pMHC-NPs-treated vs. untreated mice. Thus, treatment suppressed liver inflammation without impairing the ability of the host to clear the pathogen, presumably because the mechanisms involved in clearance of this intracellular pathogen are not impaired by TR1 cell-driven immunoregulation.

PDC-E2_166–181_/IA^g7^-NP-treated and untreated NOD.*c3c4* mice also produced similar titers of anti-dinitrophenyl (DNP) antibodies upon immunization with the hapten-carrier conjugate DNP-keyhole limpet hemocyanin (KLH) (Fig. 10e), indicating that these compounds do not impair humoral immunity against foreign antigens.

Lastly, systemic expansion and liver accumulation of PBC-suppressing PDC-E2-specific TR1-like CD4+ T-cells (Fig. 10f–n) did not impair the ability of pMHC-NP-treated NOD.*c3c4* mice to mount immune responses against allogeneic colon carcinoma (CT26) and melanoma (B16/F10) liver metastases arising upon intra-splenic injection, as compared to untreated NOD.*c3c4* mice or syngeneic hosts (Balb/c and C57BL/6J, respectively) (Fig. 10i–q).

Thus, despite targeting a systemically expressed antigen, PDC-E2_166–181_/IA^g7^-specific TR1 cells do not impair cellular or humoral immunity against local or systemic foreign antigens. This is potentially so because bacterial/viral antigenic load in local APCs transiently overwhelms the APCs’ ability to present PDC-E2 epitopes to cognate TR1-like CD4+ T-cells, hence the manifestation of their immunoregulatory properties.

## Discussion

We have used mice undergoing PBC, PSC, or AIH, as well as NSG mice humanized with PBMCs from PBC patients to investigate whether hepatocyte and/or cholangiocyte destruction in autoimmunity results in the stimulation of autoreactive T-cells capable of responding to disease-relevant and irrelevant pMHCII-based nanomedicines.

We found that nanomedicines displaying various liver-prevalent antigenic peptides triggered TR1-like cell formation and expansion in mice undergoing various liver autoimmune diseases, as well as in NSG mice humanized with PBMCs from PBC patients. As a result, these nanomedicines effectively blunted PBC, PSC, and AIH in various genetic backgrounds by suppressing liver inflammation, even when initiated at the peak of disease severity. In the liver, disease suppression involved TR1 cell-driven local Breg cell formation, required both IL10 and TGFβ, could be transferred by both tetramer+CD4+ T-cells and PCLN-associated B-cells from treated donors, and was associated with profound suppression of the pro-inflammatory capacity of both liver and liver-proximal myeloid DCs as well as Kupffer cells. In contrast, a nanomedicine displaying a pancreatic beta cell-specific epitope was unable to trigger cognate TR1 cell responses in NOD.*c3c4* mice undergoing liver autoimmunity in the absence of pancreatic autoimmunity, consistent with the sine qua non requirement for autoantigen-experience in T-cell responsiveness to pMHCII-NPs^1^.

Importantly, suppression of liver inflammation by these nanomedicines did not compromise immunity against viruses (vaccinia, influenza), intracellular bacteria (Listeria), or metastatic (liver) allogeneic tumors. The TR1-like CD4+ T-cells that are triggered by pMHC-based nanomedicines can only effect regulation when they engage cognate pMHC class II of professional APCs that are loaded with endogenous autoantigen^1^. Such APCs must capture autoantigen shed from the damaged liver cells and therefore are only present in significant numbers in the target organ or in draining lymphoid tissue. As a result, it is not surprising that pMHC-based nanomedicines do not impair immunity against systemic infections or against vaccines, as the liver-distal APCs that orchestrate these immune responses are not loaded with liver-derived autoantigens. For intra-hepatic or liver-proximal immunity, such as against a LM infection, the infected liver APCs may be overwhelmed with LM-derived antigens, decreasing their ability to elicit TR1 cell function and suppression (by dilution of cognate pMHCs at the expense of pathogen-derived pMHC complexes below the threshold required for TR1 cell activation). These cells may therefore be spared from suppression. Given the short half-lives of myeloid-derived APCs (days), replacement of these APCs by uninfected ones might be sufficient to support continued TR1-mediated immunoregulation. Alternatively, the mechanisms involved in clearance of this intracellular pathogen and allogeneic liver tumor metastases may not be impaired by TR1 cell-driven immunoregulation.

Collectively, these results have several important implications for our understanding of both normal immunity and treatment of autoimmunity. First, they demonstrate that tissue destruction in specific autoimmune diseases has the potential to trigger the stimulation or possibly outright activation of autoreactive T-cells recognizing many, perhaps all, of their antigenic components, suggesting that the antigenic repertoires in autoimmune diseases may be much more extensive than currently thought. We note that the pMHCs tested herein were designed in silico, using online MHC-binding algorithms, raising the possibility that any peptide capable of binding to self-MHC molecules, from a whole host of proteins expressed by hepatocytes and/or cholangiocytes, might be recognized by, and be able to activate peripheral T-cells. Second, our observations imply that penetrance of central and peripheral T-cell tolerance to highly expressed antigens is remarkably incomplete, even in disease-resistant genetic backgrounds. From an evolutionary standpoint, such pervasive autoreactivity may have been sustained because it functions as a source of regulatory cells capable of extinguishing pathology. Third, from a translational standpoint, this study has identified disease-modifying compounds for several complex liver autoimmune diseases that share common immunopathological pathways and represent unmet clinical needs^20,21^. Finally, these observations suggest that a few pMHCII-based nanomedicines displaying ubiquitous epitopes and HLA class II molecules encoded in oligomorphic HLA loci might be sufficient to treat various liver autoimmune diseases without impairing normal immunity.

## Methods

### Mice

NOD/LtJ, BALB/c, C57BL/6, NOD.*scid.Il2rg*^−/−^(NSG), NOD.*c3c4* and FVB/N.*Abcb4*^–/–^ (*Abcb4* or ATP-binding cassette transporter, sub-family B, member 4) mice were purchased from the Jackson Laboratory (Bar Harbor, ME). IFNγ ARE-Del^−/−^ B6 mice were obtained from H. Young (NIH, Bethesda, MD). NOD.*c3c4*.*scid* mice were generated by backcrossing (NOD.*c3c4* x NOD.*scid*) F1 mice with NOD.*c3c4* mice for five generations, followed by intercrossing of mice heterozygous for the *scid* mutation and homozygous for the B6 chromosome 3 and 4 intervals from NOD.*c3c4* mice. NOD.*Abcb4*^*–/–*^ mice were obtained by backcrossing the mutant *Abcb4* allele from FVB/N-*Abcb4*^*–/–*^ mice onto the NOD/Ltj background for six generations, followed by intercrossing. (NODxB6.IFNγ ARE-Del^–/–^) F1 mice were generated by intercrossing IFNγ ARE-Del^−/−^ and NOD/LtJ mice. NOD.*Il10*^*tm1Flv*^ (Tiger) mice were obtained by backcrossing the *Il10*^*tm1Flv*^ allele from C57BL/6.*Il10*^*tm1Flv*^ mice (Jackson Lab) onto the NOD/Ltj background for 10 generations. These studies were approved by the institutional animal care committee of the Cumming School of Medicine at the University of Calgary.

### Human subjects

HLA-DRB4*0101+ PBC patients were recruited under informed consent approved by the Institutional Ethics Review Board at Hospital Clinic (see Supplementary Table 1 for demographic and other patient details). All the work with human participants complied with all the relevant ethical regulations and was approved by the Hospital Clinic human ethics review board.

### Cell lines, pathogens, and tumors

CHO-S, BSC-1, MDCK, 293T, B16/F10, and CT26 cell lines were purchased from the ATCC (Manassas, VA). The H3N2 HKx31 and H1N1 PR8 influenza strains were from P. Thomas (St. Jude Children’s Research Hospital, Memphis, TN). LM was obtained from DMX Corporation (Philadelphia, PA).

### Antibodies and flow cytometry

FITC, PE, APC, PerCP, Alexa Fluor 647, BV605, or biotin-conjugated mAbs against mouse CD4 (RM4–5), CD5 (53–7.3), CD19 (1D3), B220 (RA36B2), CD49b (HMα2), CD25 (PC61), and CD279 (PD1; J43), anti-mCD45 (30-F11), and streptavidin–PerCP were purchased from BD Biosciences (San Diego, CA). Anti-murine LAG-3 (C9B7W) and anti-murine Foxp3 (FJK-16s) mAbs were purchased from eBioscience (San Diego, CA). Anti-latent-associated-TGF-β (LAP) (TW7–16B4), anti-CD278 (ICOS; C398.4A), anti-F4/80 (BM8), anti-hCD4 (OKT4), anti-hCD45 (HI30), and anti-mCD4 (GK1.5) antibodies were from BioLegend (San Diego, CA). PE-conjugated pMHC class II tetramers were produced using biotinylated pMHC monomers. pMHC class II tetramer staining and phenotypic marker analysis were done as follows. After avidin incubation (15 min at RT), blood leukocytes, and single cell suspensions from spleen, lymph node, liver mononuclear cells, and bone marrow cells were stained first with pMHC tetramer (5 μg ml^−1^) in FACS buffer (0.05% sodium azide and 1% FBS in PBS) for 60 min at 37 °C, and later with FITC-conjugated anti-mouse CD4 (5 μg ml^−1^) and PerCP-conjugated anti-mouse B220 (2 μg ml^−1^; as a ‘dump’ channel) for 30 min at 4 °C. After washing, cells were fixed (1% paraformaldehyde in PBS) and analyzed with FACScan, FACSaria, or BD LSRII flow cytometers. For phenotypic analyses, the cells were incubated with anti-FcR Abs, and then stained with cell surface marker antibodies diluted 1:100 in FACS buffer (at 4 °C for anti-CD49b and anti-LAP Abs, and at 37 °C for anti-LAG-3 Abs) followed by pMHC tetramer, FITC-conjugated anti-mouse CD4 (5 μg ml^−1^) and PerCP-conjugated anti-mouse B220. Upon staining, cells were washed, fixed, and analyzed by flow cytometry. FlowJo software was used for all analyses.

NSG-engrafted human T cells were analyzed using the following mAbs: FITC-conjugated anti-CD4 (OKT4, BioLegend), APC-conjugated anti-CD19 (HIB19, BD Biosciences, San Jose, CA), PerCP-conjugated polyclonal goat anti-LAG-3 IgG (R&D Systems, Minneapolis, MN), biotin-conjugated anti-CD49b (AK7, Pierce Antibodies, Thermo Fisher Scientific, Waltham, MA), and eFluor 450-conjugated streptavidin (eBioscience). Briefly, splenocytes and lymph node cells were incubated with avidin (0.25μg ml^−1^ in FACS buffer) for 30 min at room temperature, washed and stained with tetramer (5 μg ml^−1^) for 1 h at 37 °C, washed and incubated with FITC-conjugated anti-CD4 (2/100), APC-conjugated anti-CD19 (5/100; used as a ‘dump’ channel), PerCP-conjugated anti-LAG-3 (8/100), and biotin-conjugated anti-CD49b (4/100) at 4 °C for 45 min. After washing, the cells were incubated with eFluor 450-conjugated streptavidin for 30 min at 4 °C, washed, fixed in 1% PFA in PBS and cells within the hCD4^+^/hCD19^−^ gate analyzed with a FACSCanto II (BD Bioscience).

### pMHC monomers and peptides

Recombinant pMHC class II monomers were purified from supernatants of CHO-S cells transduced with lentiviruses encoding a monocistronic message in which the peptide-MHCβ and MHCα chains of the complex were separated by the ribosome skipping P2A sequence. The peptide was tethered to the amino terminal end of the MHCβ chain via a flexible GS linker and the MHCα chains were engineered encode a BirA site, a 6xHis tag, a twin strep-tag, and a free Cys at their carboxyterminal end. The secreted, self-assembled pMHC class II complexes were purified by sequential nickel and Strep-Tactin^®^ chromatography and used for coating onto NPs or processed for biotinylation and tetramer formation as described above. The epitopes encoded in the murine monomeric constructs were selected based on predicted MHCII-binding capacity using RANKPEP (http://imed.med.ucm.es/cgi-bin/rankpep_mif.cgi) using 7.54 as the threshold score. PDC-E2_166–181_ had a score that fell below the threshold but was selected for experimentation because it is contained within one of the lipoyl-binding domains of PDC-E2, an antigenic target for AMAs. For CYPD and FTCD epitope prediction, we used a second online algorithm (GPS-MBA) (http://mba.biocuckoo.org/) and peptides predicted by both RANKPEP and GPS-MBA were selected for experimentation. hPDC-E2_122–135_, hPDC-E2_249–262_ (both contained within the lipoyl-binding domain of PDC-E2), and hPDC-E2_629–643_ have been described previously (see main text). The sequences of the different epitopes are: PDC-E2_166–181_/IA^g7^ (LAEIETDKATIGFEVQ), PDC-E2_82–96_/IA^g7^ (EKPQDIEAFKNYTLD), FTCD_58–72_/IA^g7^ (VVEGALHAARTASQL), CYPD_398–412_/IA^g7^ (LITNLSSALKDETVW), 2.5mi/IA^g7^ (AHHPIWARMDA), PDC_94–108_/IA^b^ (TLDLAAAAAPQAAPA), hPDC-E2_122–135_/DRB4*0101 (GDLIAEVETDKATV), hPDC-E2_249–262_/DRB4*0101 (GDLLAEIETDKATI), and hPDC-E2_629–643_/DRB1*0801 (AQWLAEFRKYLEKPI). Synthetic PDC-E2_166–181_ and 2.5mi peptides were purchased from Genscript (Piscataway, NJ). The amino acid residue numbers for each peptide correspond to those found in the mature form of the corresponding antigens.

### NPs, pMHCII-NP synthesis, and purification

We coated pMHCs onto pegylated iron oxide NPs (PFM-NPs)^2^. PFM-NPs were produced by thermal decomposition of Fe(acac)_3_ in the presence of 2 kDa methoxy-PEG-maleimide. Briefly, 3 g Maleimide-PEG (2 kDa MW, Jenkem Tech, USA) were melted in a 50 ml round bottom flask at 100 °C and then mixed with 7 ml of benzyl ether and 2 mmol Fe(acac)_3_. The reaction was stirred for 1 h and heated to 260 °C with reflux for 2 h. The mixture was cooled to room temperature and mixed with 30 ml water. Insoluble materials were removed by centrifugation at 2000*g* for 30 min. The NPs were purified using magnetic (MACS) columns (Miltenyi Biotec, Auburn, CA) and stored in water at room temperature or 4 °C. The concentration of iron was determined spectrophotometrically at 410 nm in 2 N hydrochloric acid (HCl). Free cysteines (controls) or pMHCs, carrying a free carboxyterminal Cys, were conjugated to the maleimide-functionalized PFMs in 40 mM phosphate buffer, pH 6.0, containing 2 mM EDTA, 150 mM NaCl overnight at room temperature. The pMHC-conjugated NPs were separated from free pMHC using magnetic columns, sterilized by filtration through 0.2 µm filters and stored in water or PBS at 4 °C. Quality control was done using transmission electron microscopy, dynamic light scattering, and native and denaturing gel electrophoresis. pMHC content was measured using Bradford assay (Thermo Fisher Scientific) and SDS–PAGE.

### Purification of exhausted CD4+ and TR1-like CD4+ T-cells

Exhausted CD4+ T-cells (CD4+PD1+KLRG1+LAG3+) were FACS-sorted from the spleens of 29-week-old NOD.*c3c4* mice (*n* = 4). PDC-E2_166–181_/IA^g7^-NP-induced TR1-like CD4+ T-cells (CD4+PDC-E2_166–181_/IA^g7^-tetramer+LAG3+LAP+CD49b+) were FACS-sorted from the spleens of 22–30-week-old NOD.*c3c4* mice that had received 14 or 29 doses of PDC-E2_166–181_/IA^g7^-NPs, starting at 15 weeks of age (*n* = 1 and 3, respectively). The sorted cells were stimulated with anti-CD3/CD28 mAb-coated beads for 36 h, to measure the extent of AICD (as measured using the viability staining die 7-AAD), or for 6 days, to compare their proliferative response (by measuring BrdU incorporation using the FITC BrdU Flow kit from BD Biosciences).

### Generation of FTCD-expressing adenovirus

A replication-deficient adenovirus expressing human formiminotransferase cyclodeaminase (Ad-hFTCD) (a target autoantigen in AIH Type 2) was generated by cloning the hFTCD DNA sequence directly into Adeno-X Adenoviral System 3 (CMV) using In-Fusion^®^ HD cloning technology and Stellar Competent cells (Clontech, Mountain View, CA). Cloned Ad-FTCD was amplified in Ad-293 T cells and purified using Adeno-X Maxi Purification Kit (Clontech). The viral titer was measured using the End-point Dilution Assay or Adeno-X Rapid Titer Kit (Clontech).

### UDCA treatment

Cohorts of 5–6 or 24-week-old male and/or female NOD.*c3c4* mice were left untreated, fed a diet supplemented with 0.5% UDCA (BOC Sciences, Upton, NY; TestDiet, Richmond, IN)^22^, or treated with pMHCII-NPs for 14 or 9 weeks, respectively, and sacrificed for pMHCII tetramer staining, PBC scoring and biochemical testing.

### pMHCII-NP therapy for PBC in various genetic backgrounds

Cohorts of 15-week-old male and/or female NOD.*c3c4* mice with established PBC were left untreated or treated with 20 μg of pMHCII–NPs or Cys-NPs (i.v.) twice weekly for 9 weeks unless indicated otherwise. Liver disease scoring involved macroscopic evaluation of cyst content (0–5 for all experiments, except Fig. 6f, where cyst content was scored from 0–8), liver weight, and CBD diameter (0–4), as well as microscopic evaluation of bile duct involvement (0–4), bile duct proliferation (0–4), and mononuclear cell infiltration (0–4)^23^. In other experiments, treatment was initiated at the peak of disease (24 weeks of age) and given twice a week for 14–20 weeks. Intermittent treatment involved treating mice twice a week from 15 to 24 weeks of age, then withdrawing treatment until the percentages of tetramer+ cells dropped to ~50% of the levels seen at treatment withdrawal (measurements in peripheral blood were done once every 2 weeks), re-treating mice twice a week until the percentages of tetramer+ cells reached original values, and repeating this cycle until 50 weeks of age.

In in vivo cytokine blocking experiments, mAbs against HRPN (rIgG1), IL-10 (JES5–2A5), or TGF-β (1D11) (BioXcell, West Lebanon, NH) were given i.p. twice a week at 500 μg per dose for 2 weeks, followed by 200 μg per dose for 7 additional weeks. Mice were randomized into cytokine-neutralizing mAb-treatment (anti-IL-10 or anti-TGFβ) or HRPN rat-IgG1 groups.

In experiments involving (NOD x B6.IFNγ-ARE-Del^–/–^) F1 and B6.IFNγ-ARE-Del^–/–^ mice, 10-week-old male and female mice were treated for 5–6 weeks. Histopathologic severity in the liver was assessed by scoring the extent of portal inflammation, lobular inflammation, and granuloma formation from 0 to 4, and bile duct damage from 0 to 2. The extent of portal inflammation and bile duct damage were scored from 0 to 4 based on the ratio between affected vs. unaffected area. The extent of lobular inflammation and granuloma formation were scored from 0 to 4 based on number of lesions per specimen^14^. Inflammatory scores were obtained by adding the scores for both severity and lesion number. The severity of fibrosis was scored on a 0–6 scale as follows^24^: 0, no fibrosis; 1, fibrous expansion in few portal areas with or without small fibrous septa; 2, fibrous expansion in most portal areas with or without small fibrous septa; 3, fibrous expansion in most portal areas with very few portal-to-portal bridging; 4, fibrous expansion in all portal areas with marked bridging (portal-to-portal and portal-to-central); 5, marked bridging with very few nodules (incomplete cirrhosis); and 6, complete cirrhosis.

Studies using NOD mice involved treating cohorts of 10-week-old pre-diabetic female NOD/Ltj mice with 20 μg of pMHCII-NPs or Cys-NPs i.v. twice weekly for 5 weeks.

### pMHCII-NP therapy for PSC in NOD.*Abcb4*^*–/–*^ mice

Cohorts of 5–6-week-old male and/or female NOD.*Abcb4*^*–/–*^ mice with established PSC^25^ were treated with 20 μg of pMHCII-NPs or Cys-NPs i.v. twice weekly for 5–6 weeks. Histopathologic lesions were graded using the Ishak scoring system^24,26^, which evaluates both fibrosis (0–6), as well as necroinflammatory sequelae of biliary cholangitis, including interface hepatitis (0–4), confluent necrosis (0–6), lobular inflammation (0–4), and portal inflammation (0–4).

### pMHCII-NP therapy for AIH in NOD mice

We induced AIH by infecting 5–6-week-old female NOD/Ltj mice with an adenovirus encoding human FTCD (Ad-hFTCD, 10^10^ plaque forming units (PFU) i.v.), as previously described^19^. Four weeks later, cohorts of mice with established AIH were treated with 20 μg of pMHCII-NP s or Cys-NPs (i.v.) twice weekly for 5–6 weeks. Histopathological scoring was done using the Ishak scale as above^24,26^.

### pMHCII-NP therapy in human PBMC-reconstituted NSG hosts

PBMCs from HLA-DRB4*0101+ PBC patients (recruited under informed consent approved by the Institutional Review Board at Hospital Clinic) were depleted of CD8+ T-cells (to reduce the magnitude of GvHD in the hosts) using anti-CD8 mAb-coated magnetic beads (Miltenyi Biotech, Auburn, CA) and injected i.v. (2 × 10^7^) into 8–10-week-old NSG hosts. Mice were treated with 30–40 μg pMHC-NPs starting on day 5 after PBMC transfusion, twice a week for 5 consecutive weeks, or left untreated. Therapy-induced expansion of cognate CD4+ T-cells was measured in liver, peripheral LNs, and spleen (Supplementary Table 1). The gender, age, anti-mitochondrial autoantibody status, and type of pMHC-NP tested for each patient are summarized in Supplementary Table 1. A mouse was considered a responder if the percentage of tetramer+ T-cells in at least two different organs were higher than the mean ± 10 standard deviation values seen in untreated hosts.

### Evaluation of general adaptive immunity

Evaluation of cellular responses to Vaccinia infection was performed as previously described^1^. Briefly, pMHCII-NP-treated and untreated female mice were injected i.v. with 2 × 10^6^ PFU of recombinant Vaccinia Virus (rVV) and sacrificed on days 4 and 14 after infection. Samples were processed for pMHCII tetramer staining and rVV titer measurements. Briefly, both ovaries were collected in DMEM containing 2% FBS, homogenized, freeze-thawed three times followed by sonication (three rounds, 20 s each). Serial dilutions of the lysates were added to confluent BSC-1 cell cultures at 37 °C for 1 h, washed twice with serum-free DMEM and then overlaid with DMEM containing 2% FCS and 0.4% carboxymethyl cellulose (CMC; Sigma, Saint Louis, MO). On day 3, the overlay was discarded, and the cell layers were stained with crystal violet to count the number of plaques.

To evaluate cellular responses to Influenza infection, pMHCII-NP-treated and untreated mice were first primed i.p. with the HKx31 (H3N2) strain at 10^6^ EID_50_ per mouse. One cohort of mice was sacrificed 7 days after priming and processed for tetramer staining to confirm presence of pMHC-NP-specific TR1-like cells during priming. Other cohorts of primed mice were re-infected 30 days later with an intranasal dose of PR8 virus, a lethal H1N1 strain of Influenza (8 × 10^4^ EID_50_ per mouse), under anesthesia. PR8-challenged mice were weighed daily and scored clinically from 0 to 4 based on the extent of ruffled fur, reduced motility, huddled appearance, and rapid and/or labored breathing as previously described^27^. Mice were sacrificed 7 days later and processed for tetramer staining and influenza titer measurement. Briefly, lungs were collected in serum-free DMEM, homogenized and freeze-thawed three times. Serial dilutions of the lysates were added to confluent MDCK cell cultures at RT for 1 h and washed. Cultures were then overlaid with DMEM containing 0.4% CMC and TPCK-trypsin for 2–3 days, washed, fixed, and stained with crystal violet to count plaques.

Cellular immunity to intracellular bacteria was determined by infecting pMHCII-NP-treated and untreated mice i.v. with 10^3^ cfu of LM. In some experiments, mice were sacrificed 7 days or 14 days after infection and samples processed for tetramer staining and bacterial load measurements. Briefly, spleen and liver were cut into several pieces, weighted and homogenized in PBS containing 0.35% Triton X-100. Serial dilutions of the lysates were then plated onto Bovine Heart Infusion agar containing 5 μg ml^−1^ erythromycin, incubated for 24–48 h at 37 °C and the number of colonies counted. In other experiments, mice were infected with LM immediately before the initiation of pMHC-NP therapy and sacrificed on day 3 to confirm presence of LM cfu in both liver and spleen, or on day 35, after termination of treatment (two doses/week for 5 weeks).

Cellular immunity to liver metastatic tumors was ascertained upon intra-splenic injections of B16/F10 melanoma and CT26 colon carcinoma tumors into syngeneic (C57BL/6J or Balb/c, respectively) or allogeneic hosts (pMHC-NP-treated or untreated NOD.*c3c4* mice)^28,29^. A small incision was made in the abdomen, under isofluorane inhalational anesthesia, to partially expose the spleen. Tumor cells (0.2 × 10^6^ and 0.1 × 10^6^ for B16/F10 and CT26, respectively, in 100 μl of PBS) were injected slowly for 1 min into the exposed spleen. Ten minutes later, the spleen was removed and the peritoneal and skin layers sutured. pMHCII-NP therapy was resumed within 5–7 days after surgery and continued until the end of follow-up. Mice were monitored for up to 19–21 days and euthanized for tetramer staining, PBC scoring, and tumor burden measurements. In B16/F10-injected mice, tumor burden was assessed by measuring liver weight and counting the number of metastases, easily distinguishable from liver parenchyma. In CT26-injected animals, tumor burden was scored histologically by measuring the hepatic area (HPA) occupied by metastatic tumors.

To evaluate humoral immunity, pMHCII-NP-treated and untreated mice were immunized i.p. with 100 μg of DNP-KLH (Alpha Diagnostic International, San Antonio, TX) in CFA and boosted again 3 weeks later as previously described^1^. Mice were sacrificed 10 days later, to measure serum anti-DNP antibody titers using an anti-DNP Ig ELISA Kit (Alpha Diagnostic International).

### Cytokine secretion assays

Splenic and portal/celiac lymph node (PCLN) cell suspensions from pMHCII-NP-treated mice were enriched for CD4+ T-cells depleting CD19+ B-cells (EasySep™ Mouse CD19 Positive Selection Kit, Stem Cell Technologies, Vancouver, BC) and CD8+ T-cells (CD8 Magnetic Particles, BD Biosciences). Cells were stained with pMHCII tetramers and sorted by flow cytometry. The sorted cells (2–3 × 10^4^) were challenged with bone marrow-derived DCs (2 x10^4^) pulsed with 2 μg ml^−1^ peptide. Forty-eight hours later, supernatants were harvested for measurement of cytokine content via Luminex^®^.

To ascertain whether pMHCII-NP therapy promoted the recruitment/formation of IL-10-secreting B-cells, mesenteric LNs, PCLNs, and liver cell suspensions were enriched for B-cells using a CD19 enrichment kit (Stem Cell Technologies). The cells (2–3 × 10^5^ in 200 μl/well) were stimulated in duplicate with LPS (1 μg ml^−1^, Sigma) for 24 h in RPMI-1640 media containing 10% FCS. The levels of IL-10 in the supernatants were measured via Luminex^®^.

### CD11b+ cells and Kupffer cells

CD11b+ cells from LNs were obtained by digestion in collagenase D (1.25 µg ml^−1^) and DNAse I (0.1 µg ml^−1^) for 15 min at 37 °C, washed, incubated with anti-FcR Abs, and purified using anti-CD11b mAb-coated magnetic beads (BD Biosciences). The purified cells (2–3 × 10^5^ in 200 μl/well) were stimulated with LPS (2 μg ml^−1^) for 3 days, and the supernatants analyzed for cytokine content using a Luminex^®^ multiplex cytokine assay.

To isolate Kupffer cells (KCs), livers from treated and untreated mice were minced and digested in 15 ml of 0.05% collagenase solution in HBSS for 20–30 min at 37 °C. The resulting cell suspension was filtered through a nylon mesh (0.7 μm) and centrifuged at 50×*g* for 3 min at 4 °C, to remove tissue debris and hepatocytes. Cells in the supernatant were pelleted by centrifugation at 300×*g* for 5 min at 4 °C. The cell pellet, mainly composed of non-parenchymal liver immune cells, KCs, sinusoidal endothelial cells, and stellate cells, was re-suspended in 33% Percoll^®^ solution and centrifuged at 350×*g* for 30 min to isolate mononuclear cells. The pellets were re-suspended in DMEM containing 10% FCS (5 × 10^6^ cells ml^−1^) and plated in six-well plate at 1–3 × 10^7^ cells/well and incubated for 2–3 h in a 5% CO_2_ atmosphere at 37 °C. Non-adherent cells were removed by gentle washing with PBS. The adherent fraction (enriched for KCs) was harvested by trypsin digestion (5 min, 0.25% trypsin). The resulting cell suspension was plated in 96-well plates at 1–2 × 10^5^/200 μl/well and stimulated with LPS (2 μg ml^−1^) for 3 days. The supernatants were analyzed for cytokine content using a Luminex^®^ multiplex cytokine assay.

### Adoptive transfer of suppression

Splenic CD4+ T-cells (10^7^) from untreated mice or mice treated with 12 doses of PDC-E2_166–181_/IA^g7^-NPs were adoptively transferred (i.v.) into 10–14-week old, sex-matched NOD.*c3c4.scid* hosts. One day later, the recipients were adoptively transferred with 4 × 10^7^ whole splenocytes from sex-matched NOD.*c3c4* donor mice with established PBC (>35-week old). One of the cohorts of mice transfused with CD4+ T-cells from pMHCII-NP-treated donors was further treated with 12 doses of PDC-E2_166–181_/IA^g7^-NPs. The recipients were sacrificed 6 weeks later for tetramer staining and PBC scoring.

In other experiments, the hosts were transfused with 5 × 10^5^ CD19+ cells purified from the PCLNs or PLNs of mice treated with 10 doses of PDC-E2_166–181_/IA^g7^-NPs or BDC2.5 mi/IA^g7^-NPs, respectively, during the preceding 5 weeks. B-cells were purified using the EasySep Mouse CD19-positive selection Kit II (StemCell Technologies, Vancouver, BC). Other cohorts received PDC-E2_166–181_/IA^g7^ or BDC2.5mi/IA^g7^ tetramer + (2 × 10^5^) T-cells FACS-sorted from the spleen and liver or pancreas-draining lymph nodes of PDC-E2_166–181_/IA^g7^-NP- or BDC2.5mi/IA^g7^-NP-treated donors, respectively.

### In vivo Breg induction assay

Splenic B-cells from NOD.*Il10*^*tm1Flv*^ (Tiger) mice were enriched using an EasySep Mouse B-cell Isolation Kit (Stem Cell Technologies) and pulsed with BDC2.5mi or PDC_166–181_ peptides (10 μg ml^−1^) for 2 h at 37 °C as previously described^1^. The peptide-pulsed B-cells were washed twice with PBS, labeled with PKH26 (Sigma) and transfused (3 × 10^6^) into pMHC-NP-treated or untreated mice. The hosts were killed 7 days later and their spleens, MLNs, PCLNs, and liver mononuclear cells were labeled with anti-B220-APC and biotinylated anti-CD1d or anti-CD5 mAbs followed by Streptavidin-PerCP. PKH26+ B-cells were analyzed for presence of eGFP+/CD1d^high^ and eGFP+/CD5+ cells by flow cytometry.

### Histology and immunohistochemistry

Livers were fixed in 10% formalin for 2 days, embedded in paraffin, cut into 5 μm sections and stained with H&E or Picrosirius Red. We scored (~0.5 cm^2^) sections from the four liver lobes from each mouse (right and left, median and caudal) and a minimum of four portal triads per lobe section (16 portal triads/mouse). For immunohistochemistry, liver tissues were embedded in Tissue-Tek OCT, sectioned into 30 µm cryosections and stored on slides at −80 °C. Slides were fixed in chilled acetone, washed with PBS, treated with a 1:10 dilution of 30% H_2_O_2_ in PBS, washed with PBS, blocked with 10% normal goat serum in PBS, washed again, and stained with anti-mouse CD4 (GK1.5) or CD8 (Lyt-2) antibodies (1.5 h, 4 °C). After washing, the slides were stained with a biotinylated goat anti-rat secondary antibody (1:200 dilution), incubated with horseradish peroxidase (HRP)-conjugated streptavidin, followed by 3,3-diaminobenzidine (DAB) substrate. Slides were counterstained with hematoxylin before mounting.

### ALT and TBA assays

ALT levels in serum were determined using a kit from Thermo Fisher Scientific following the manufacturer’s protocol. Briefly, serum samples were mixed with pre-warmed (37 °C) InfinityTM ALT (GPT) Liquid Stable Reagent at 1:10 ratio and OD readings were taken for 3 min at 1 min intervals in a nanodrop at a 340 nm wavelength, 37 °C. The slope was calculated by plotting absorbance vs. time using linear regression and multiplied with a factor to obtain ALT levels in serum (U/l) as described in the kit. Serum TBA levels were analyzed using a TBA Enzymatic Cycling Assay Kit (Diazyme, Poway, CA) following the manufacturer’s protocol but using 96-well plates instead of cuvettes^14^.

### Anti-nuclear and anti-mitochondrial autoantibodies

Presence of ANAs in serum was ascertained using NOVA Lite^®^HEp-2 Slides kit (Inova Diagnostics, San Diego, CA). A semi-quantitative approach was followed to measure ANA titers. Briefly, serum samples were serially diluted in PBS (at 1:160, 1:320, 1:640, 1:1280, and 1:2560) and then added to pre-fixed Hep-2 substrate slides, washed, stained with FITC-conjugated goat anti-mouse IgG in PBS containing 5% normal donkey serum (1:200 dilution), washed, mounted, and read under a fluorescent microscope.

Serum levels of anti-mitochondrial PDC-E2 antibodies were determined via ELISA. Briefly, ELISA plates were coated with PDC-E2 protein (5 μg ml^−1^, 100 μl) (SurModics Inc, Eden Prairie, MN) overnight at RT. Plates were washed, blocked using 3% dry skim milk in PBS (pH 7.4, 150 μl), and incubated with serially diluted serum samples (100 μl, at 1:250 dilutions prepared using reagent diluent) for 2 h at RT. Wells were washed and incubated with 100 μl of HRP-conjugated anti-mouse IgG (1:2000 in reagent diluent) for 2 h at RT, and washed. Finally, wells were incubated in the dark with 100 μl of DAB substrate for 20 min at RT. Upon stopping the enzymatic reaction with 50 μl 2 N H_2_SO_4_, the absorption was measured at a 450 nm wavelength using an ELISA plate reader. The positive antibody activity (PAA) levels were calculated by calculating the mean OD ± 2 SD of the control NOD serum samples (positive index) and by dividing the OD values corresponding to NOD.*c3c4* serum samples by the positive index, whereby values > 1.0 correspond to PAA.

### Statistical analyses

Unless specified, sample size values mentioned in the figure legends correspond to the total number of mice examined, pooled from different experiments. Data were compared in GraphPad Prism 6 using Mann–Whitney *U*-test, Kruskal–Wallis test, Chi-Square, Log-Rank (Mantel–Cox), Pearson correlation, two-way ANOVA or multiple *t*-test analyses using the Holm–Sidak correction. *P*-values < 0.05 were considered statistically significant. Only statistically significant *P* values are displayed in figures.

### Reporting summary

Further information on research design is available in the Nature Research Reporting Summary linked to this article.

## Supplementary information

Supplementary File

Reporting Summary

## Data Availability

Raw data used to generate the figures of the manuscript are available from the authors upon request.
