# Supplementary material for: Suppression of a broad spectrum of liver autoimmune pathologies by single peptide-MHC-based nanomedicines
Source: Nat Commun. 2019 May 14;10:2150. doi: 10.1038/s41467-019-09893-5 (PMC6517389; doi:10.1038/s41467-019-09893-5)
Supplement: Supplementary file 1 — Supplementary File [file 41467_2019_9893_MOESM1_ESM.pdf]

SUPPLEMENTARY INFORMATION

UMESHAPPA ET AL.,

SUPPRESSION OF A BROAD SPECTRUM OF LIVER AUTOIMMUNE PATHOLOGIES BY SINGLE PEPTIDE-MAJOR HISTOCOMPATIBILITY COMPLEX-BASED NANOMEDICINES

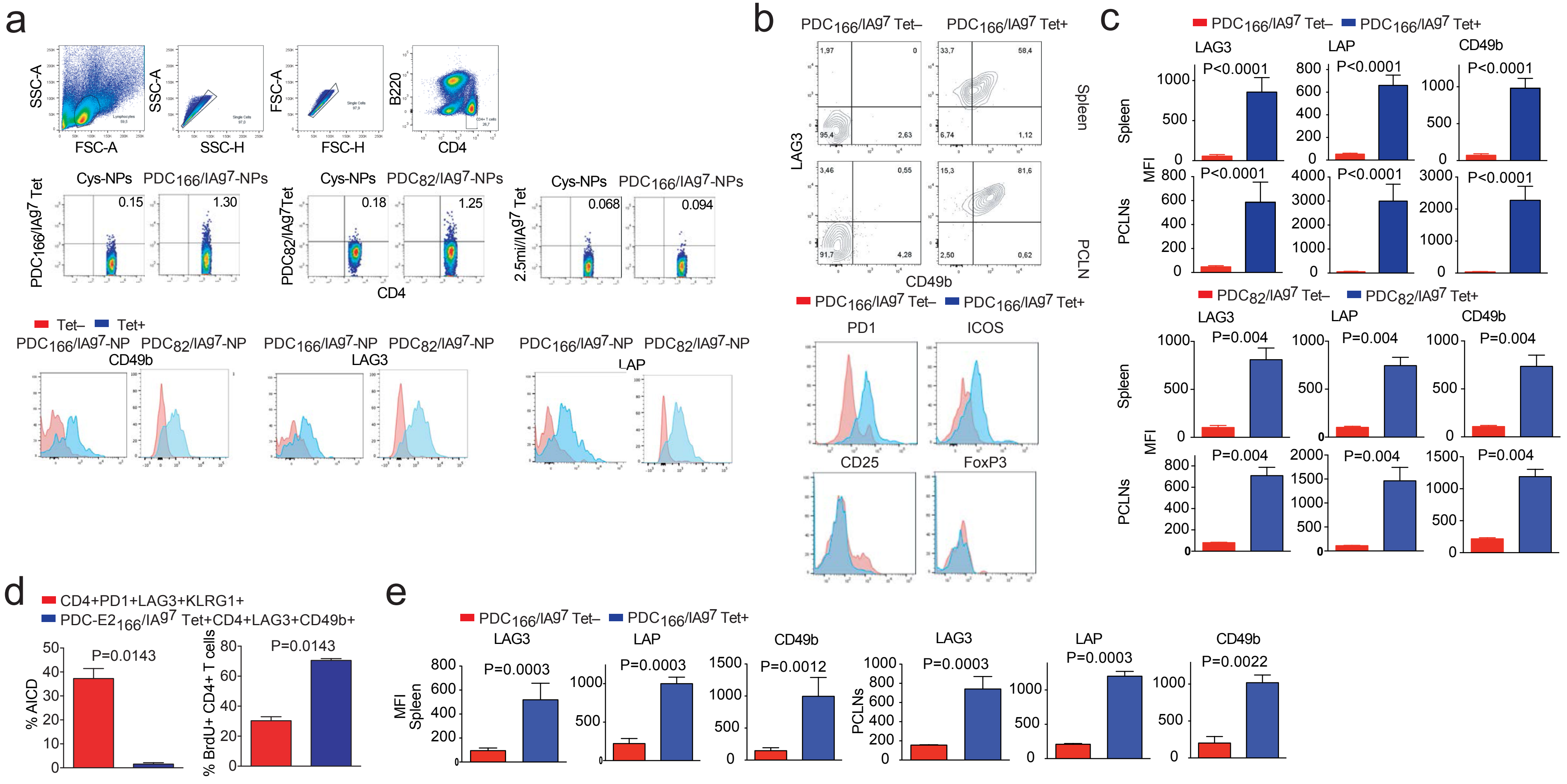
a
SSC-A
FSC-A
SSC-A
SSC-H
FSC-A
FSC-H
B220
CD4
Cys-NPs
PDC166/IAg7-NPs
PDC166/IAg7 Tet
0.15
1.30
Cys-NPs
PDC82/IAg7-NPs
PDC82/IAg7Tet
0.18
1.25
CD4
Cys-NPs
PDC166/IAg7-NPs
2.5mi/IAg7 Tet
0.068
0.094
Tet–
Tet+
PDC166/IAg7-NP
PDC82/IAg7-NP
CD49b
PDC166/IAg7-NP
PDC82/IAg7-NP
LAG3
PDC166/IAg7-NP
PDC82/IAg7-NP
LAP
b
PDC166/IAg7 Tet–
PDC166/IAg7 Tet+
LAG3
CD49b
Spleen
PCLN
1,97
0
95,4
2,63
33,7
58,4
6,74
1,12
3,46
0,55
91,7
4,28
15,3
81,6
2,50
0,62
PDC166/IAg7 Tet–
PDC166/IAg7 Tet+
PD1
ICOS
CD25
FoxP3
c
PDC166/IAg7 Tet–
PDC166/IAg7 Tet+
LAG3
LAP
CD49b
Spleen
PCLNs
MFI
P<0.0001
P<0.0001
P<0.0001
P<0.0001
P<0.0001
P<0.0001
PDC82/IAg7 Tet–
PDC82/IAg7 Tet+
LAG3
LAP
CD49b
Spleen
PCLNs
MFI
P=0.004
P=0.004
P=0.004
P=0.004
P=0.004
P=0.004
d
CD4+PD1+LAG3+KLRG1+
PDC-E2166/IAg7 Tet+CD4+LAG3+CD49b+
% AICD
P=0.0143
% BrdU+ CD4+ T cells
P=0.0143
e
PDC166/IAg7 Tet–
PDC166/IAg7 Tet+
MFI
Spleen
PCLNs
LAG3
P=0.0003
LAP
P=0.0003
CD49b
P=0.0012
LAG3
P=0.0003
LAP
P=0.0003
CD49b
P=0.0022

Supplementary Figure 1. **PBC-relevant pMHCII-NPs expand TR1-like CD4+ T-cells**.

a-c, Representative lymphocyte gating strategy and tetramer stains used to obtain all tetramer staining values in the manuscript **(a, top)** and upregulation of TR1-like markers on tetramer+CD4+ T-cells expanded *in vivo* in response to $PDC_{166\text{-}181}/IA^{g7}$- or $PDC_{82\text{-}96}/IA^{g7}$-NP therapy (**a, bottom,** showing representative LAG3, LAP and CD49b histograms for spleen-associated tetramer+ vs. tetramer– T-cells**; b top,** showing representative two-dimensional LAG3/CD49b contour plots for splenic and PCLN-associated tetramer+ vs. tetramer– T-cells**; b bottom**, showing histograms for CD25, ICOS and PD1 for PCLN-associated tetramer+ vs. tetramer– cells**;** and **c,** showing average mean fluorescence intensity values for LAG3, LAP and CD49b for splenic and PCLN tetramer+ vs. tetramer– cells from $PDC_{166\text{-}181}/IA^{g7}$- or $PDC_{82\text{-}96}/IA^{g7}$-NP-treated mice). Data in a and c correspond to 8-10 mice/organ. Data in b correspond to n=3 (top) and n=4 (bottom). **d,** Percentage of exhausted CD4+PD1+KLRG1+LAG3+ cells from 29 wk-old untreated NOD.*c3c4* mice or percentage of pMHC-NP-induced $PDC_{166\text{-}181}/IA^{g7}$ tetramer+ CD4+LAG3+LAP+CD49b+ cells from 22-30 wk-old NOD.*c3c4* mice treated with 14 or 29 doses of $PDC\text{-}E2_{166\text{-}181}/IA^{g7}$-NPs starting at 15 wk of age (n=1 and 3, respectively), that undergo activation-induced cell death (AICD) or incorporate BrdU upon stimulation with anti-CD3/anti-CD28 mAb-coated microparticles *ex vivo* (for 36h or 6 days, respectively). Data correspond to n=4/cell type. **e**, Average mean fluorescence intensity values for TR1 cell surface markers on tetramer+CD4+ T-cells arising in 38-44 wk-old NOD.*c3c4* mice in response to $PDC_{166\text{-}181}/IA^{g7}$-NP therapy. Data correspond to 7 mice/organ. Data correspond to the mean $\pm$ SEM. P values were compared via Mann-Whitney U.

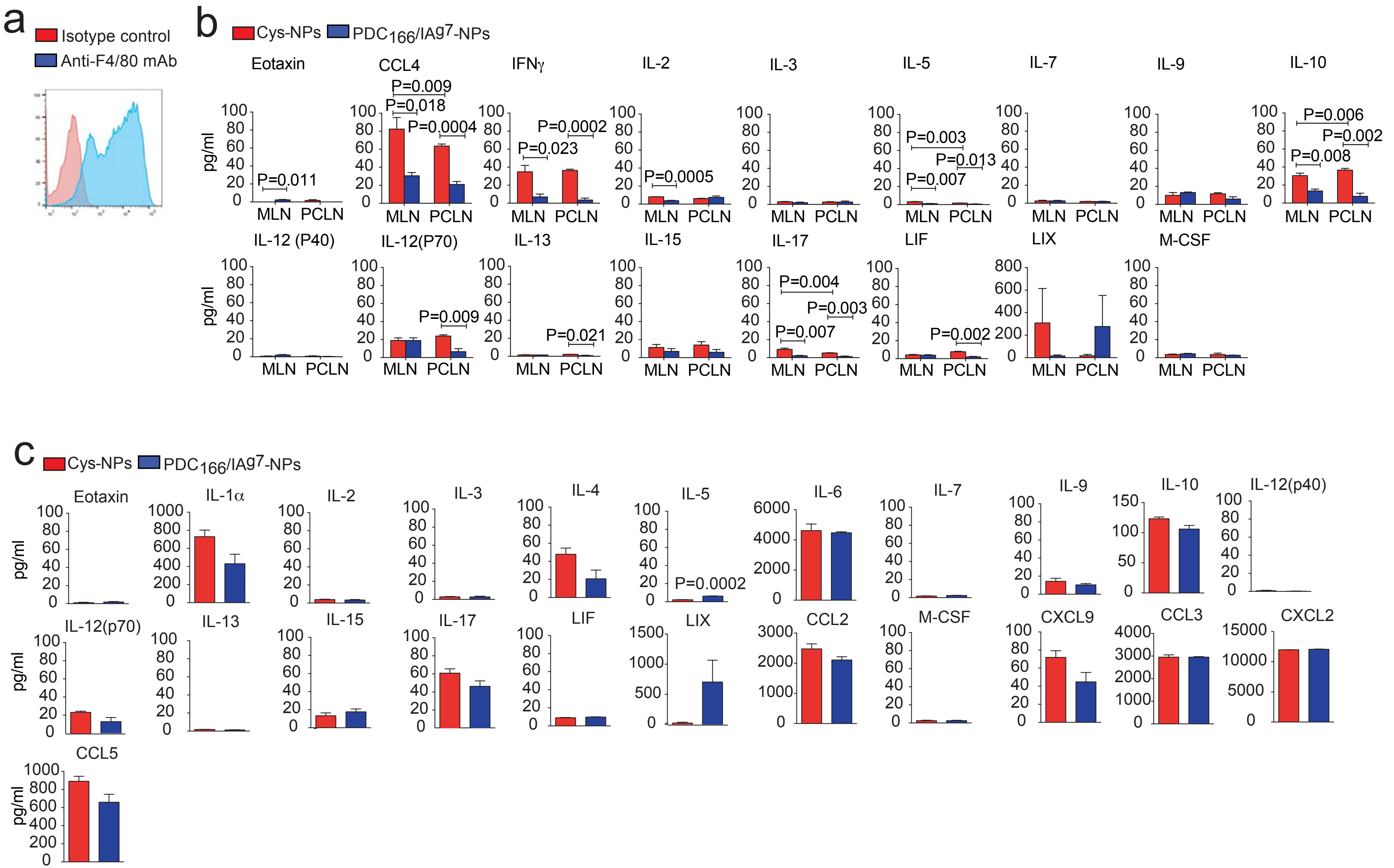
a
Isotype control
Anti-F4/80 mAb
b
Cys-NPs
PDC166/IAg7-NPs
Eotaxin
CCL4
IFNγ
IL-2
IL-3
IL-5
IL-7
IL-9
IL-10
pg/ml
P=0.011
P=0.009
P=0.018
P=0.0004
P=0.023
P=0.0002
P=0.0005
P=0.003
P=0.013
P=0.007
P=0.006
P=0.002
P=0.008
MLN
PCLN
IL-12 (P40)
IL-12(P70)
IL-13
IL-15
IL-17
LIF
LIX
M-CSF
P=0.009
P=0.021
P=0.004
P=0.003
P=0.007
P=0.002
c
Cys-NPs
PDC166/IAg7-NPs
Eotaxin
IL-1α
IL-2
IL-3
IL-4
IL-5
IL-6
IL-7
IL-9
IL-10
IL-12(p40)
P=0.0002
IL-12(p70)
IL-13
IL-15
IL-17
LIF
LIX
CCL2
M-CSF
CXCL9
CCL3
CXCL2
CCL5
pg/ml

Supplementary Figure 2. Cytokine profiles of LPS-challenged PCLN and MLN-derived CD11b+ cells and Kupffer cells. a, Histogram overlay for anti-F4/80 vs. isotype control mAb staining of purified Kupffer cells. Purity ranged from 75-89%. **b and c**, Cytokine secretion profiles for PCLN vs. MLN-derived CD11b+ cells **(b)** or liver Kupffer **(c)** from NOD.*c3c4* mice treated with Cys- or $PDC_{166\text{-}181}/IA^{g7}$-NP. Only cytokines/chemokines that do not show statistically significant differences between PCLN and MLN (**for b**) or between Cys- or $PDC_{166\text{-}181}/IA^{g7}$-NP-treated mice (**for c**) are shown here (those showing statistically significant differences are displayed in main Fig. 5).

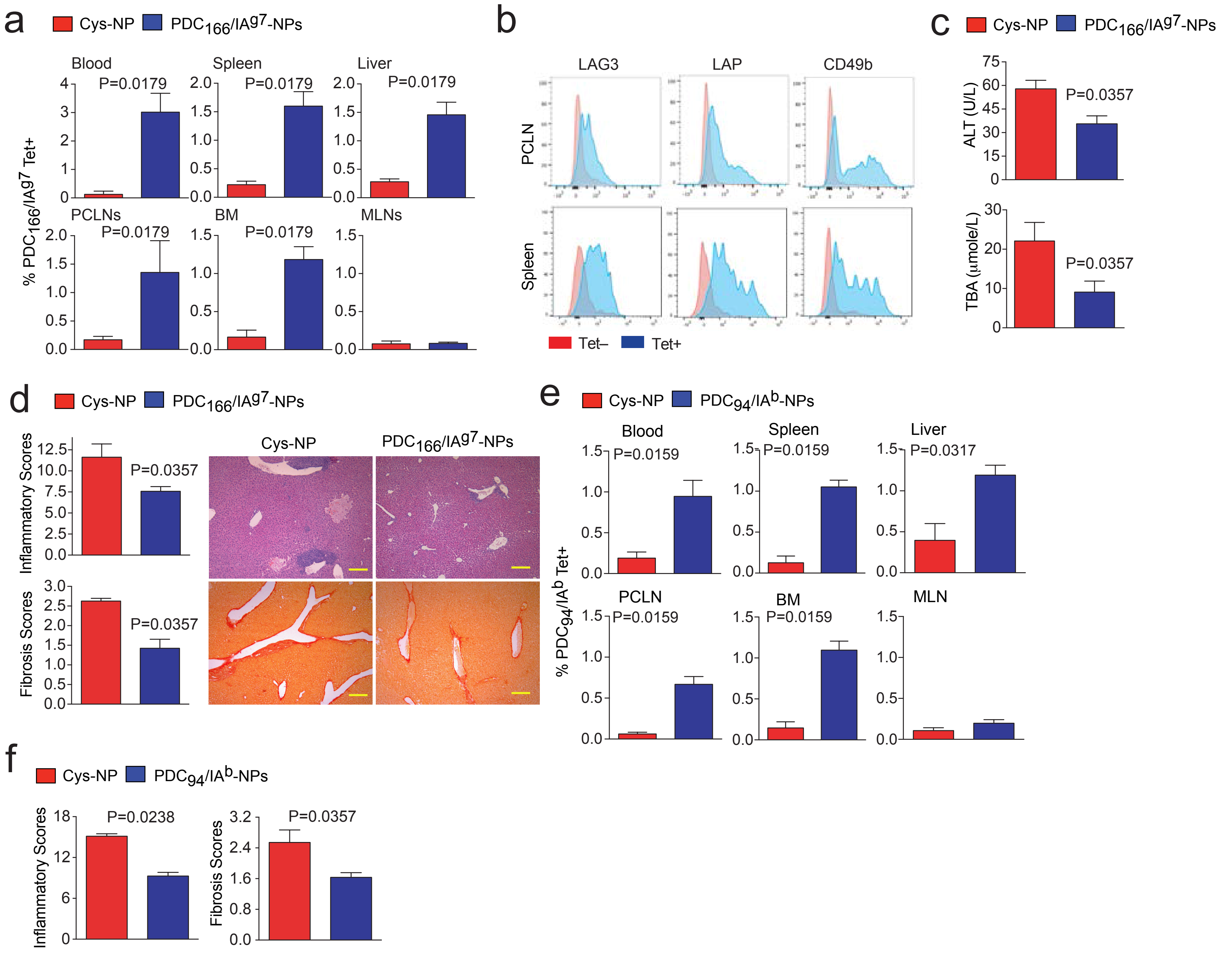

a
Cys-NP
PDC166/IAg7-NPs
Blood
Spleen
Liver
PCLNs
BM
MLNs
P=0.0179
% PDC166/IAg7 Tet+
b
LAG3
LAP
CD49b
PCLN
Spleen
Tet–
Tet+
c
ALT (U/L)
P=0.0357
TBA (µmole/L)
d
Cys-NP
PDC166/IAg7-NPs
Inflammatory Scores
Fibrosis Scores
P=0.0357
e
Cys-NP
PDC94/IAb-NPs
Blood
Spleen
Liver
PCLN
BM
MLN
P=0.0159
P=0.0317
% PDC94/IAb Tet+
f
Cys-NP
PDC94/IAb-NPs
Inflammatory Scores
Fibrosis Scores
P=0.0238
P=0.0357

Supplementary Figure 3. Therapeutic effects of PBC-relevant pMHCII-NPs in (NODxB6.*Ifng*-ARE-Del$^{-/-}$) F1 and B6.*Ifng*-ARE-Del$^{-/-}$mice.

a, Percentages of tetramer+ CD4+ T-cells in female (NODxB6.*Ifng*-ARE-Del$^{-/-}$) F1 mice treated with Cys-NPs or $PDC_{166\text{-}181}/IA^{g7}$-NPs. **b**, Representative LAG3, LAP and CD49b staining profiles for tetramer+ vs. tetramer– CD4+ T-cells. **c,** Serum TBA and ALT levels in the mice studied in a. **d,** Microscopic scores of the mice studied in a (top) and representative H&E and Picrosirius Red-stained liver sections (bottom). Scale bars: 100 μm. Data in a-c correspond to n=5 and 3 mice/treatment type, respectively, from 2 experiments. Similar results were obtained in males (n=7 and 4/treatment type, respectively). **e and f**, Percentages of tetramer+ CD4+ T-cells (e) and microscopic liver pathology scores (f) in female B6.*Ifng*-ARE-Del$^{-/-}$mice treated with Cys-NPs or $PDC_{94\text{-}108}/IA^{b}$-NPs. Data correspond to n=3 and 6 mice/treatment type, respectively.

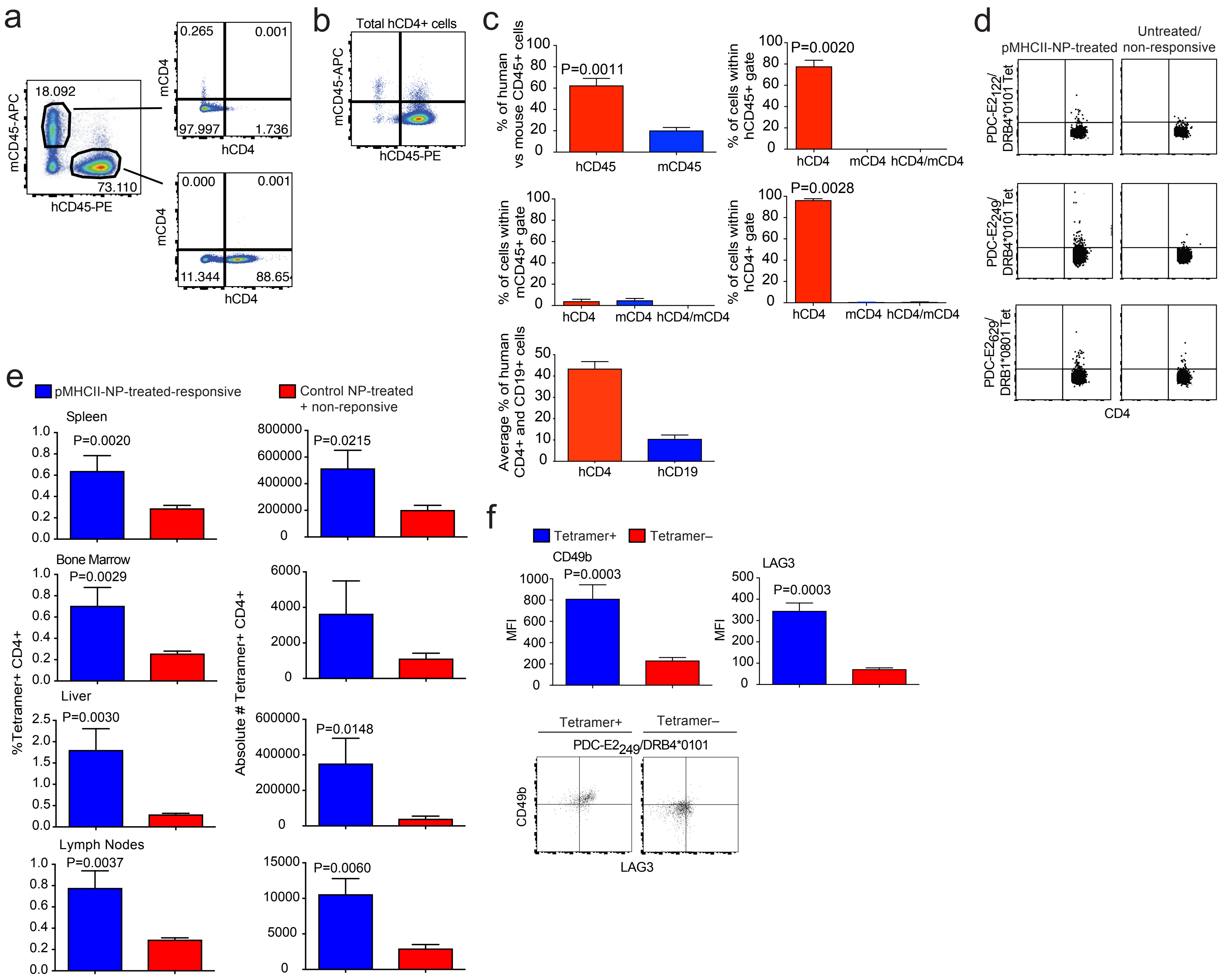

a
mCD45-APC
hCD45-PE
18.092
73.110
mCD4
hCD4
0.265
0.001
97.997
1.736
0.000
0.001
11.344
88.65
b
Total hCD4+ cells
mCD45-APC
hCD45-PE
c
% of human vs mouse CD45+ cells
P=0.0011
hCD45
mCD45
% of cells within hCD45+ gate
P=0.0020
hCD4
mCD4
hCD4/mCD4
% of cells within mCD45+ gate
% of cells within hCD4+ gate
P=0.0028
Average % of human CD4+ and CD19+ cells
hCD4
hCD19
d
pMHCII-NP-treated
Untreated/ non-responsive
PDC-E2122/ DRB4*0101 Tet
PDC-E2249/ DRB4*0101 Tet
PDC-E2629/ DRB1*0801 Tet
CD4
e
pMHCII-NP-treated-responsive
Control NP-treated + non-reponsive
Spleen
P=0.0020
P=0.0215
Bone Marrow
P=0.0029
Liver
P=0.0030
P=0.0148
Lymph Nodes
P=0.0037
P=0.0060
%Tetramer+ CD4+
Absolute # Tetramer+ CD4+
f
Tetramer+
Tetramer–
CD49b
P=0.0003
LAG3
P=0.0003
MFI
PDC-E2249/DRB4*0101
CD49b
LAG3

Supplementary Figure 4. **Pharmacodynamic effects of PBC-relevant pMHCII-NPs in NSG mice engrafted with PBMCs from PBC patients.**

a-b, Representative flow cytometry plots for splenocytes from human PBMC-engrafted NSG hosts. **a**, Anti-mCD45/hCD45 plots and mCD4/hCD4 plots from gated mCD45+/hCD45– and mCD45–/hCD45+ cells, respectively. **b**, mCD45/hCD45 plots from gated hCD4+ cells. **c**, Average percentages of hCD45+, mCD45+, hCD4+, mCD4+ and hCD19+ cells. Data in c correspond to 6 (top four plots) and 30 mice (bottom plot), respectively. **d**, Representative tetramer stains in NSG hosts engrafted with PBMCs from DRB4*0101+ PBC patients and treated with three different human PBC-relevant pMHCII-NPs. **e**, Average percentages and absolute numbers of tetramer+ CD4+ cells in responsive (n=11) vs. unresponsive pMHCII-NP-treated mice (n=6) plus untreated littermates (n=34). **f**, Mean fluorescence intensity values (top) and two-dimensional FACS plots (bottom) for the TR1 markers CD49b and LAG3 in the mice studied in **d-e**. Data correspond the mean ± SEM. P values were compared via Mann-Whitney U, except the top four panels of c (Kruskal-Wallis test).

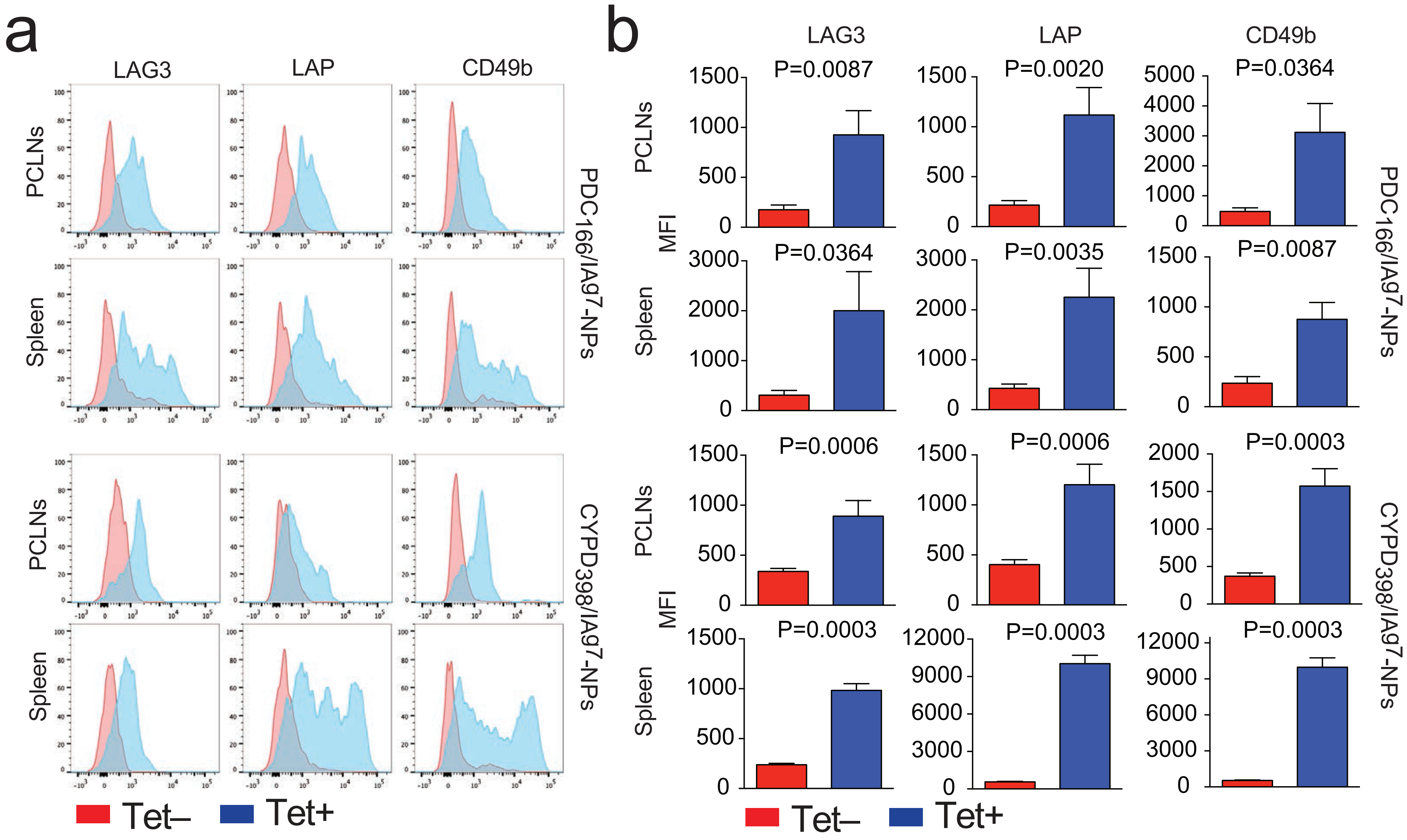

a
LAG3
LAP
CD49b
PCLNs
Spleen
PCLNs
Spleen
PDC166/IA97-NPs
CYPD398/IA97-NPs
Tet–
Tet+
b
LAG3
LAP
CD49b
P=0.0087
P=0.0020
P=0.0364
P=0.0364
P=0.0035
P=0.0087
P=0.0006
P=0.0006
P=0.0003
P=0.0003
P=0.0003
P=0.0003
MFI
MFI
PCLNs
Spleen
PCLNs
Spleen
PDC166/IA97-NPs
CYPD398/IA97-NPs
Tet–
Tet+

Supplementary Figure 5. **PBC- and AIH-relevant pMHCII-NPs expand TR1-like cells in mice with PSC.**

a-b, Representative FACS staining histograms (**a**) and average mean fluorescence intensity values for TR1 markers on tetramer+CD4+ vs. tetramer-CD4+ T-cells (**b**) of NOD.*Abcb4*$^{-/-}$ mice treated with PBC- ($PDC_{166-181}$/IA^{g7}-NP) or AIH-relevant pMHCII-NPs ($CYPD_{398-412}$/IA^{g7}-NPs). Data correspond to n=7 mice/group from 2 experiments.

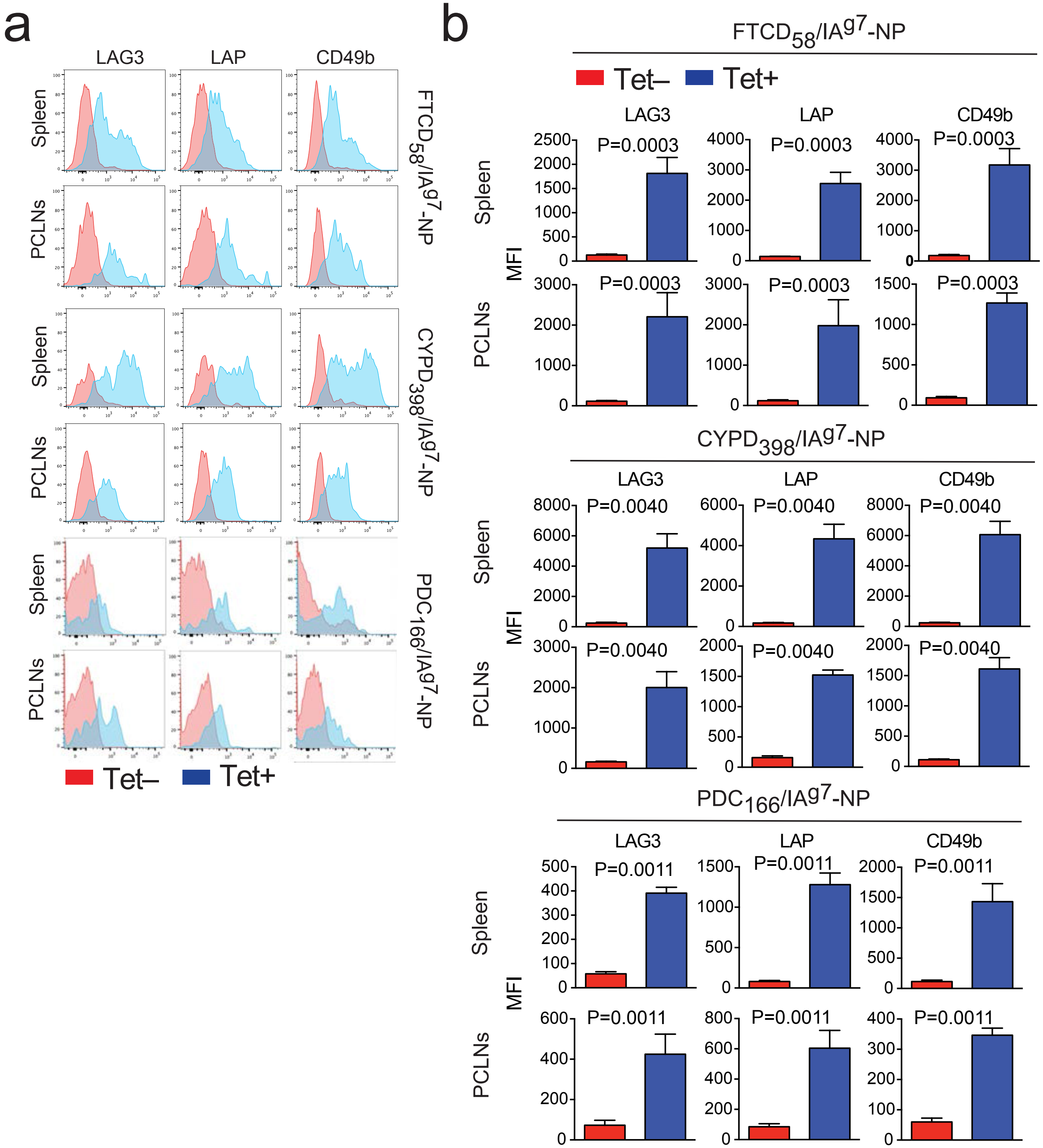
a
LAG3
LAP
CD49b
Spleen
PCLNs
FTCD58/IAg7-NP
CYPD398/IAg7-NP
PDC166/IAg7-NP
Tet–
Tet+
b
FTCD58/IAg7-NP
CYPD398/IAg7-NP
PDC166/IAg7-NP
MFI
P=0.0003
P=0.0040
P=0.0011

Supplementary Figure 6. **PBC- and AIH-relevant pMHCII-NPs expand TR1-like cells and blunt disease in mice with AIH.**

a-b, Representative FACS staining histograms (**a**) and average mean fluorescence intensity values for TR1 markers on tetramer+CD4+ vs. tetramer- CD4+ T-cells (**b**) of NOD mice infected with Ad-hFTCD upon treatment with AIH- ($FTCD_{58-72}/IA^{g7}$-NP or $CYPD_{398-412}/IA^{g7}$-NP) or PBC-relevant pMHCII-NPs ($PDC_{166-181}/IA^{g7}$-NP). Data correspond to n=7, 5 and 6 mice, respectively, from 2 experiments). Data in B were compared by Mann-Whitney U.

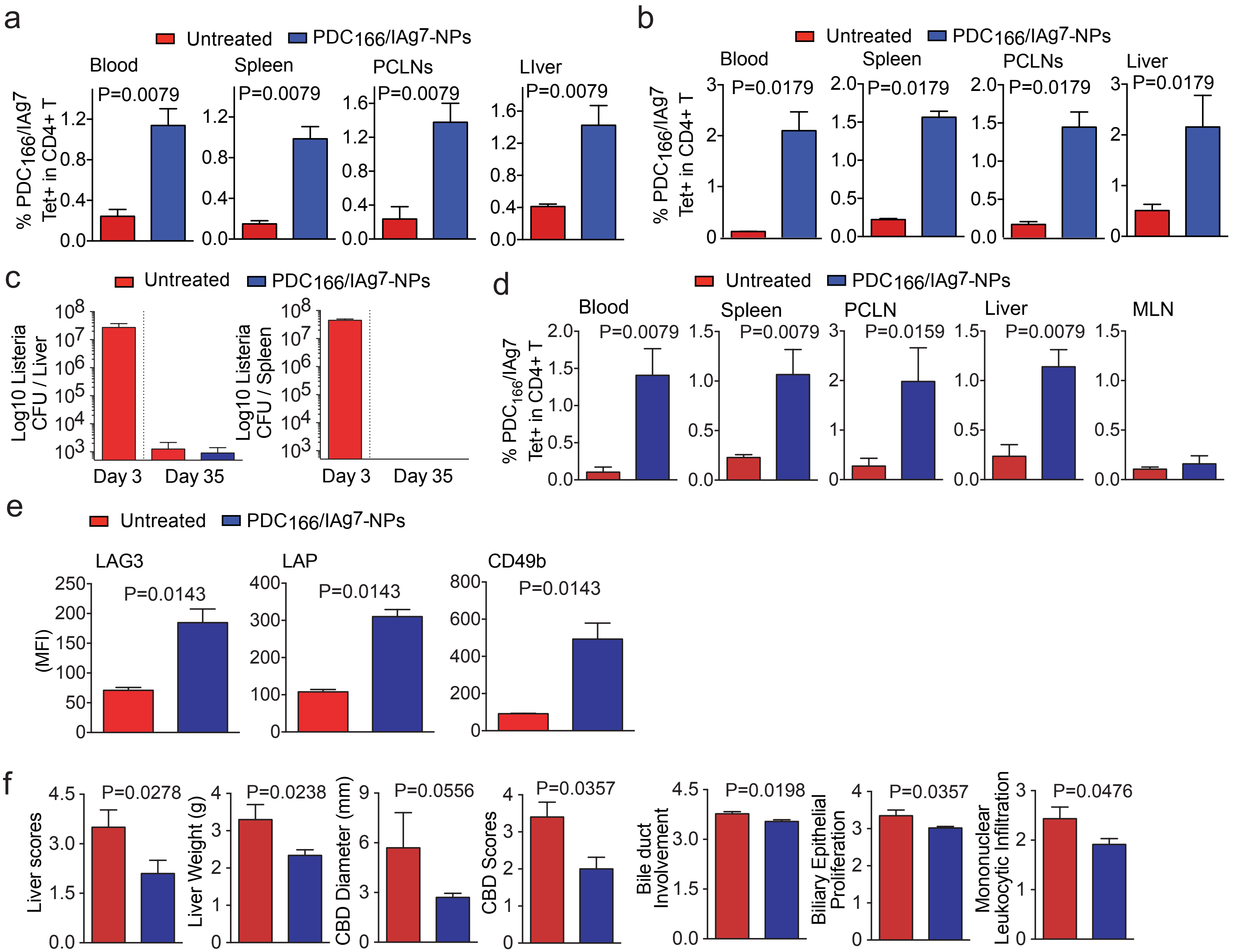

a
Untreated
PDC166/IAg7-NPs
Blood
Spleen
PCLNs
LIver
P=0.0079
% PDC166/IAg7 Tet+ in CD4+ T
b
Untreated
PDC166/IAg7-NPs
Blood
Spleen
PCLNs
Liver
P=0.0179
% PDC166/IAg7 Tet+ in CD4+ T
c
Untreated
PDC166/IAg7-NPs
Log10 Listeria CFU / Liver
Log10 Listeria CFU / Spleen
Day 3
Day 35
d
Untreated
PDC166/IAg7-NPs
Blood
Spleen
PCLN
Liver
MLN
P=0.0079
P=0.0159
% PDC166/IAg7 Tet+ in CD4+ T
e
Untreated
PDC166/IAg7-NPs
LAG3
LAP
CD49b
P=0.0143
(MFI)
f
P=0.0278
P=0.0238
P=0.0556
P=0.0357
P=0.0198
P=0.0476
Liver scores
Liver Weight (g)
CBD Diameter (mm)
CBD Scores
Bile duct Involvement
Biliary Epithelial Proliferation
Mononuclear Leukocytic Infiltration

Supplementary Figure 7. **Disease-relevant pMHCII-NPs spare general immunity despite triggering the formation and expansion of PDC-E2 autoreactive TR1-like CD4+ T-cells**.

a, Average percentages of $PDC_{166\text{-}181}/IA^{g7}$ tetramer+CD4+ T-cells in blood and lymphoid organs of $PDC_{166\text{-}181}/IA^{g7}$-NP-treated NOD.*c3c4* mice 7 days after priming with HKx31 strain. Data correspond to n=4 and 5, respectively. **b**, Average percentages of $PDC_{166\text{-}181}/IA^{g7}$ tetramer+CD4+ T-cells in blood and lymphoid organs of $PDC_{166\text{-}181}/IA^{g7}$-NP-treated NOD.*c3c4* mice 14 days after infection with *L. monocytogenes*. Data correspond to n=3 and 5 mice, respectively. **c**, Colony-forming units of *L. monocytogenes* in the spleen and liver of untreated vs. $PDC_{166\text{-}181}/IA^{g7}$-NP-treated NOD.*c3c4* mice 3 and 35 days after infection. Data correspond to n=2 (untreated), 5 (untreated) and 5 (treated) mice from left to right, respectively. **d,e**, Average percentages of (d), and MFI for CD49b, Lag3 and LAP (e) of $PDC_{166\text{-}181}/IA^{g7}$ tetramer+CD4+ T-cells in lymphoid organs of untreated and $PDC_{166\text{-}181}/IA^{g7}$-NP-treated NOD.*c3c4* mice 35 days after infection with *L. monocytogenes*. **f**, Liver weights and macroscopic/microscopic liver disease scores for the mice studied in c-e. Data in c-f correspond to n=5 and 4-5 mice/treatment type and read-out, respectively. Data correspond to the mean $\pm$ SEM. P values were compared via Mann-Whitney U.

hPDC-E2(122-135)-DRB4-PFM-010616 (30mcg/dose). DRB4*01:01 patients										
ID	Gender	Age (yr)	AMAs	Spleen		Liver		LN		Outcome
				Treated % hCD4 Number of cells	Untreated % hCD4 Number of cells	Treated % hCD4 Number of cells	Untreated % hCD4 Number of cells	Treated % hCD4 Number of cells	Untreated % hCD4 Number of cells	
B013	F	79	+	0.055	0.303	2.288	0.302	0.863 (A)	0.274	+
				63756	141561	300444	39069	23760	154	
B009	F	76	+	1.253	0.291	0.199	0.307	ND		+
				732729	308949	7047	11853	ND		
B005	M	74	+	No engraftment						
B001	M	59	+	0.319	0.296	1.625	0.296	1.082 (A)	0.312	+
				186539	291863	256354	62871	28524	1874	
B021	F	68	+	0.767	0.309	0.343	0.296	0.728 (A)	0.300	+
				849816	509331	18691	28993	15072	5721	
B011	F	71	+	0.169	0.304	0.091	0.294	0.462 (A)	0.291	–
				202383	209370	6658	151772	120	755	
B018	F	56	+	Dead						
B012	F	71	+	0.709	0.298			0.722 (A)	0.290 (A)	+
				341920	124050	ND		5602	2101	

hPDC-E2(249-262)-DRB4-PFM-010616 (40mcg/dose). DRB4*01:01 patients										
ID	Gender	Age (yr)	AMAs	Spleen		Liver		LN		Outcome
				Treated % hCD4 Number of cells	Untreated % hCD4 Number of cells	Treated % hCD4 Number of cells	Untreated % hCD4 Number of cells	Treated % hCD4 Number of cells	Untreated % hCD4 Number of cells	
B013	F	79	+	0.078	0.310	4.925	0.295	0.621 (PC)	0.245	+
				105174	164589	1124257	35712	33121	275	
B009	F	76	+	0.028	0.307	0.515	0.297	ND		–
				31807	192099	20215	10866	ND		
B004	F	63	+	0.427	0.294	0.094	0.315	0.089 (A)	0.293	+
				268565	228575	2990	299574	526	11251	
B012	F	70	+	0.264	0.297	0.078	0.300	0.059 (PC)	0.313	–
				41467	147568	1034	24584	1513	6160	
B001	M	59	+	Dead						
B021	F	68	+	1.612	0.310	1.943	0.289	2.589 (PC)	0.320	+
				1368420	514802	1174100	35690	9491	788	
B019	F	73	+	Dead						
B014	F	72	+	0.709	0.295	0.588	0.305	1.096 (A)	0.299	+
				604820	162521	76559	30198	5557	1271	

hPDC-E2(629-643)-DR8-PFM-010616 (30mcg/dose). DRB1*08:01 patients										
ID	Gender	Age (yr)	AMAs	Spleen		Liver		LN		Outcome
				Treated % hCD4 Number of cells	Untreated % hCD4 Number of cells	Treated % hCD4 Number of cells	Untreated % hCD4 Number of cells	Treated % hCD4 Number of cells	Untreated % hCD4 Number of cells	
B022	F	72	+	0.024	0.300	0.619	0.293	No cells	0.295 (PC)	–
				662	48190	26	29984		260	
B021	F	68	+	0.725	0.317	3.185	0.300	1.103 (A)	0.301	+
				1095456	562089	455716	36134	22752	8080	
B020	F	70	+	0.303	0.308	0.180	0.297	No cells		–
				38207	114160	19765	46701			
B025	F	69	+	0.404	0.308	2.895	0.294	0.501 (A)	0.319 (A)	+
				59552	49721	78551	8747	740	466	
B023	F	75	+	0.745	0.292	0.283	0.292	0.371 (A)	0.297	–
				416501	181855	4960	4517	3740	2232	

Supplementary Table 1. **Percentages and absolute numbers of tetramer+CD4+ T-cells in NSG mice engrafted with PBMCs from DRB4*0101+ PBC patients, upon treatment with three different human PBC-relevant pMHCII-NP types**. ND, not determined. PC, portal and celiac. A, axylar. See Methods for scoring criteria.
