# Supplementary material for: Suppression of a broad spectrum of liver autoimmune pathologies by single peptide-MHC-based nanomedicines
Source: Nat Commun. 2019 May 14;10:2150. doi: 10.1038/s41467-019-09893-5 (PMC6517389; doi:10.1038/s41467-019-09893-5)
Supplement: Supplementary file 2 — Reporting Summary [file 41467_2019_9893_MOESM2_ESM.pdf]

nature research

Corresponding author(s): Pere Santamaria

Reporting Summary

Nature Research wishes to improve the reproducibility of the work that we publish. This form provides structure for consistency and transparency in reporting. For further information on Nature Research policies, see Authors & Referees and the Editorial Policy Checklist.

Statistical parameters

When statistical analyses are reported, confirm that the following items are present in the relevant location (e.g. figure legend, table legend, main text, or Methods section).

n/a	Confirmed	
☐	☒	The exact sample size (*n*) for each experimental group/condition, given as a discrete number and unit of measurement
☐	☒	An indication of whether measurements were taken from distinct samples or whether the same sample was measured repeatedly
☐	☒	The statistical test(s) used AND whether they are one- or two-sided<br>*Only common tests should be described solely by name; describe more complex techniques in the Methods section.*
☒	☐	A description of all covariates tested
☐	☒	A description of any assumptions or corrections, such as tests of normality and adjustment for multiple comparisons
☐	☒	A full description of the statistics including central tendency (e.g. means) or other basic estimates (e.g. regression coefficient) AND variation (e.g. standard deviation) or associated estimates of uncertainty (e.g. confidence intervals)
☐	☒	For null hypothesis testing, the test statistic (e.g. *F*, *t*, *r*) with confidence intervals, effect sizes, degrees of freedom and *P* value noted<br>*Give P values as exact values whenever suitable.*
☒	☐	For Bayesian analysis, information on the choice of priors and Markov chain Monte Carlo settings
☒	☐	For hierarchical and complex designs, identification of the appropriate level for tests and full reporting of outcomes
☒	☐	Estimates of effect sizes (e.g. Cohen's *d*, Pearson's *r*), indicating how they were calculated
☐	☒	Clearly defined error bars<br>*State explicitly what error bars represent (e.g. SD, SE, CI)*

Our web collection on statistics for biologists may be useful.

Software and code

Policy information about availability of computer code

Data collection	FlowJo, Prism 7
Data analysis	Prism 7, SSPS Statistics

For manuscripts utilizing custom algorithms or software that are central to the research but not yet described in published literature, software must be made available to editors/reviewers upon request. We strongly encourage code deposition in a community repository (e.g. GitHub). See the Nature Research guidelines for submitting code & software for further information.

Data

Policy information about availability of data

All manuscripts must include a data availability statement. This statement should provide the following information, where applicable:

- Accession codes, unique identifiers, or web links for publicly available datasets
- A list of figures that have associated raw data
- A description of any restrictions on data availability

Raw data used to build the figures are available upon request

Field-specific reporting

Please select the best fit for your research. If you are not sure, read the appropriate sections before making your selection.

☒ Life sciences ☐ Behavioural & social sciences ☐ Ecological, evolutionary & environmental sciences

For a reference copy of the document with all sections, see nature.com/authors/policies/ReportingSummary-flat.pdf

Life sciences study design

All studies must disclose on these points even when the disclosure is negative.

Sample size	Based on previous studies in similar biological systems. Sample sizes were as large as possible with tight controls of gender, age, dosing regiments, and experimental conditions within and between experiments, to limit experimental variability. In vitro experiments typically involved smaller sample sizes than in vivo experiments and all statistically significant differences are reported with the corresponding P values.
Data exclusions	No data/mice were excluded from analyses. All analyzed samples are reported.
Replication	Most data sets were replicated in independent experiments and the number of experiments that were pursued are specifically stated in the Figure legends . In addition, the manuscript used several redundant disease model systems (e.g. similar disease but in a different genetic background) that further substantiate the robustness of the conclusions.
Randomization	Randomization into individual treatment groups was pre-determined. Mice were entered into the study when they reached a pre-determined disease score that ensured sustained disease progression and disease chronicity in all the mice. This information is provided in Methods and Figure legends.
Blinding	Investigators were not blinded to study outcome, but were generally replicated by different investigators. All cytokine/chemokine determinations were blinded.

Reporting for specific materials, systems and methods

Materials & experimental systems

n/a	Involved in the study	
☐	☒	Unique biological materials
☐	☒	Antibodies
☐	☒	Eukaryotic cell lines
☒	☐	Palaeontology
☐	☒	Animals and other organisms
☐	☒	Human research participants

Methods

n/a	Involved in the study	
☒	☐	ChIP-seq
☐	☒	Flow cytometry
☒	☐	MRI-based neuroimaging

Unique biological materials

Policy information about availability of materials

Obtaining unique materials	Unique materials (e.g. pMHC monomers, tetramers) are available from the authors upon request. If required amounts exceed our ability to supply, we will provide detailed protocols.

Antibodies

Antibodies used	All antibodies were obtained form commercial sources and the clone numbers and source identified in the Online Methods section.
Validation	Validation was from the commercial supplier and/or the published scientific literature using the exact same antibodies.

Eukaryotic cell lines

Policy information about cell lines

Cell line source(s)	Source of all cell lines and cell line identity are indicated within the manuscript.

April 2018

Authentication	CHO cells were commercially available and were not authenticated in our laboratories upon receipt. They were used for pMHC production. However, we regularly sequence transduced pMHC-production cell lines (the transduced genes), to confirm identity in terms of pMHC specificity.
Mycoplasma contamination	Cell lines used for pMHC production were free of mycoplasma contamination.
Commonly misidentified lines (See ICLAC register)	N/A

Animals and other organisms

Policy information about studies involving animals; ARRIVE guidelines recommended for reporting animal research

Laboratory animals	Mouse gender and age are available in the figure legends, tables and/or methods. All studies were approved by the corresponding institutional animal care committees.
Wild animals	N/A
Field-collected samples	N/A

Human research participants

Policy information about studies involving human research participants

Population characteristics	The demographic information on the patient population that was sampled is described in the results and materials and methods section as well as in TableS1. All patients signed an informed consent form and were informed of the objectives of the study before samples were collected. The consent form was approved by the IDIBAPS Institutional Review Board.
Recruitment	Patients were recruited into providing samples for HLA typing by the attending physician during a regular visit. Only patients carrying the HLA alleles of interest were recruited for further studies (in NSG mice humanized with PBMC samples).

Flow Cytometry

Plots

Confirm that:

☒ The axis labels state the marker and fluorochrome used (e.g. CD4-FITC).

☒ The axis scales are clearly visible. Include numbers along axes only for bottom left plot of group (a 'group' is an analysis of identical markers).

☒ All plots are contour plots with outliers or pseudocolor plots.

☒ A numerical value for number of cells or percentage (with statistics) is provided.

Methodology

Sample preparation	This is described in the Methods section.
Instrument	The instruments used are indicated in the Methods section.
Software	FlowJo
Cell population abundance	Where useful, we provide absolute cell number values (e.g. in Table S1, for humanized NSG mice; or Fig. 3d, showing a corrleation between tetramer+ T-cell and B-cell content in draining vs non-draining lymph nodes).
Gating strategy	All samples were gated equally, by identifying the lymphocyte gate in the forward/side scatter plot, excluding cell aggregates, measuring presence of tetramer+ cells within the CD4+B220– gate, using identical gates for control and pMHC-NP-treated mice. Fig. S1a provides representative tetramer stains to illustrate this.

☒ Tick this box to confirm that a figure exemplifying the gating strategy is provided in the Supplementary Information.
